# Impact of CaSO_4_-rich soil on Miocene surface preservation and Quaternary sinuous to meandering channel forms in the hyperarid Atacama Desert

**DOI:** 10.1038/s41598-022-22787-9

**Published:** 2022-10-26

**Authors:** Benedikt Ritter, Julia L. Diederich-Leicher, Steven A. Binnie, Finlay M. Stuart, Volker Wennrich, Andreas Bolten, Tibor J. Dunai

**Affiliations:** 1 Institute of Geology & Mineralogy, University of Cologne, Cologne, Germany; 2 Isotope Geosciences Unit, Scottish Universities Environmental Research Centre, East Kilbride, UK; 3 Institute of Geography, University of Cologne, Cologne, Germany

**Keywords:** Palaeoclimate, Geochemistry, Geomorphology, Tectonics

## Abstract

The Atacama Desert is the driest and oldest desert on Earth. Despite the abundance evidence for long-term landscape stability, there are subtle signs of localised fluvial erosion and deposition since the onset of hyperaridity in the rock record. In the dry core of the Atacama Desert, pluvial episodes allowed antecedent drainage to incise into uplifting fault scarps, which in turn generated sinuous to meandering channels. Incision of ancient alluvial fan surfaces occurred during intermittent fluvial periods, albeit without signs of surface erosion. Fluvial incision during predominantly hyperarid climate periods is evident from these channels in unconsolidated alluvium. The absence of dense vegetation to provide bank stability and strength led us to investigate the potential role of regionally ubiquitous CaSO_4_-rich surface cover. This has enabled the preservation of Miocene surfaces and we hypothesize that it provided the required bank stability by adding strength to the upper decimetre to meter of incised alluvium to allow high sinuosity of stream channels to form during pluvial episodes in the Quaternary.

## Introduction

Hyperarid conditions have prevailed in the dry core of the Atacama Desert since the Early Miocene^1–4^. There are several inter-related reasons for the aridification; (1) its position within the area of the subtropical high pressure belt^5^, (2) upwelling of the cold Humboldt current in conjunction with the evolution and existence of a persistent atmospheric inversion layer trapping moist Pacific air below 1000 m a. s. l.^6,7^, (3) the orogenic rainshadow effect of the Andes to the east^5–8^, as well as (4) the continental rainshadow effect of moisture originating from the Atlantic. The timing and location of the onset of aridity, and its evolution are still a matter of debate. For instance, there are strong indications that parts of the hyperarid Coastal Cordillera and Central Depression (19–23°S) experienced the earliest onset of hyperaridity during the Early Miocene^1,2,4^. Long-term hyperarid conditions and the absence of significant precipitation enabled the evolution of thick surface covers of evaporites and dust, sourced from atmospheric deposition^9–11^. Relict surfaces are covered by decimetre to meter thick calcium-sulphate (CaSO_4_) rich soils or colluvium^11^ and are unable to sustain significant macroscopic vegetation. However, there is evidence that the predominantly hyperarid conditions were repeatedly interrupted by wetter—though still (hyper-) arid—periods^2^ which were capable of initiating fluvial incision and erosion^2,3,12^.

**Figure 1:**
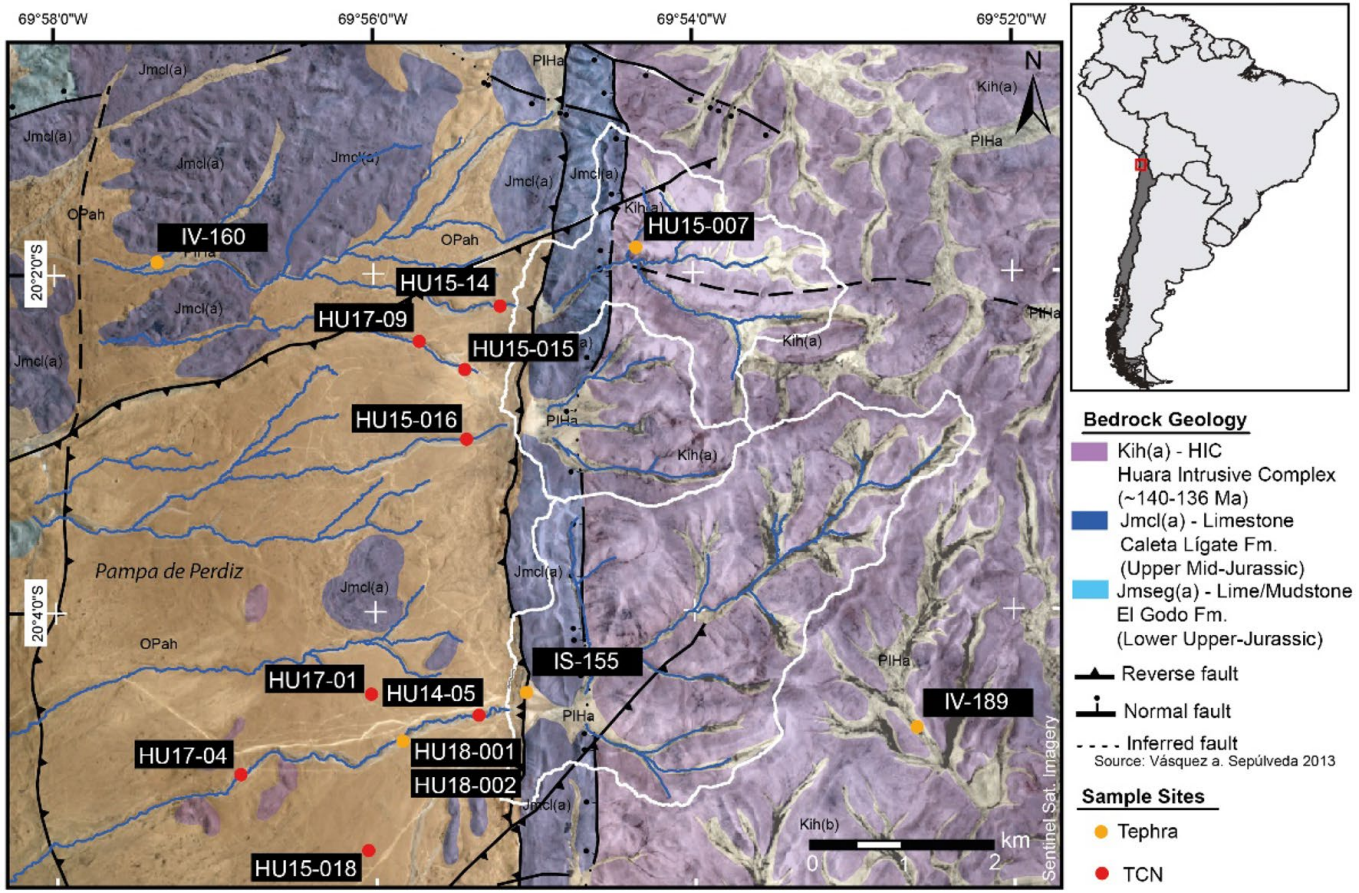
Overview map of the study area based on Sentinel 1B satellite imagery (created using ArcGIS Pro 2.8.1—pro.arcgis.com—and Adobe Illustrator 2022—adobe.com). Blue lines represent inactive fluvial channels, colour shading indicate bedrock geology. Tectonic faults are from Vásquez and Sepúlveda^19^. All surfaces are covered by a decimeter to meter thick CaSO_4_-rich surface cover, especially flat alluvial surface in the Pampa de Perdiz. White contours highlight the three endorheic catchments, that were formerly the headwaters for the channels investigated. The western escarpment of the HIC is characterized by a combination of a NS reverse and normal faults, causing the exhumation of Jurassic rocks and the tectonic damming of westward directed drainages of the HIC. Orange dots mark locations of tephra deposits (own sites have the prefix HU, the others are from^19^). Sampling sites for TCN exposure dating are marked with red dots.

Fluvial systems in arid-hyperarid environments are characterized by large alluvial fans and small incised channels^13^, as well as braided channel networks, where ephemeral drainage dominates^13,14^. They form in response to rare, but intense precipitation events^15^. Ephemeral streams are often unstable and their channel form quickly alter in response to precipitation events, which cause a dynamic expression by channel migration, low temporal stability, high gradients and mostly a predominance of bedload^15,16^. Assuming that pluvial periods in the Atacama Desert are ephemeral, the observation of single thread sinuous to meandering channels in the hyperarid core of the Atacama Desert is surprising (Fig. 1). Sinuous to meandering channel forms are reported in modern poorly vegetated arid environments^16,17^, however, only rarely in ancient hyperarid environments^16–18^, such as the core of the Atacama Desert. This suggests that they are either fossil, formed during wetter periods prior, or the development of high sinuosity fluvial systems was enabled by other processes.

In this study we bracket the timing of alluvial fan abandonment and channel incision in the Atacama Desert using cosmogenic nuclide exposure dating of quartz clasts (^10^Be, ^26^Al and ^21^Ne) and tephrochronology. This frames the paleoenvironmental conditions during channel formation and provides causal mechanism for the development of sinuous to meandering stream channels in a hyperarid environment. We show that fluvial incision was coeval with long term (hyper-) aridity and that the ubiquitous CaSO_4_-rich soil and crust helped to preserve landscapes and promoted high sinuosity of fluvial channels.

## Alluvial fans and meander in the hyperarid Atacama Desert

**Figure 2:**
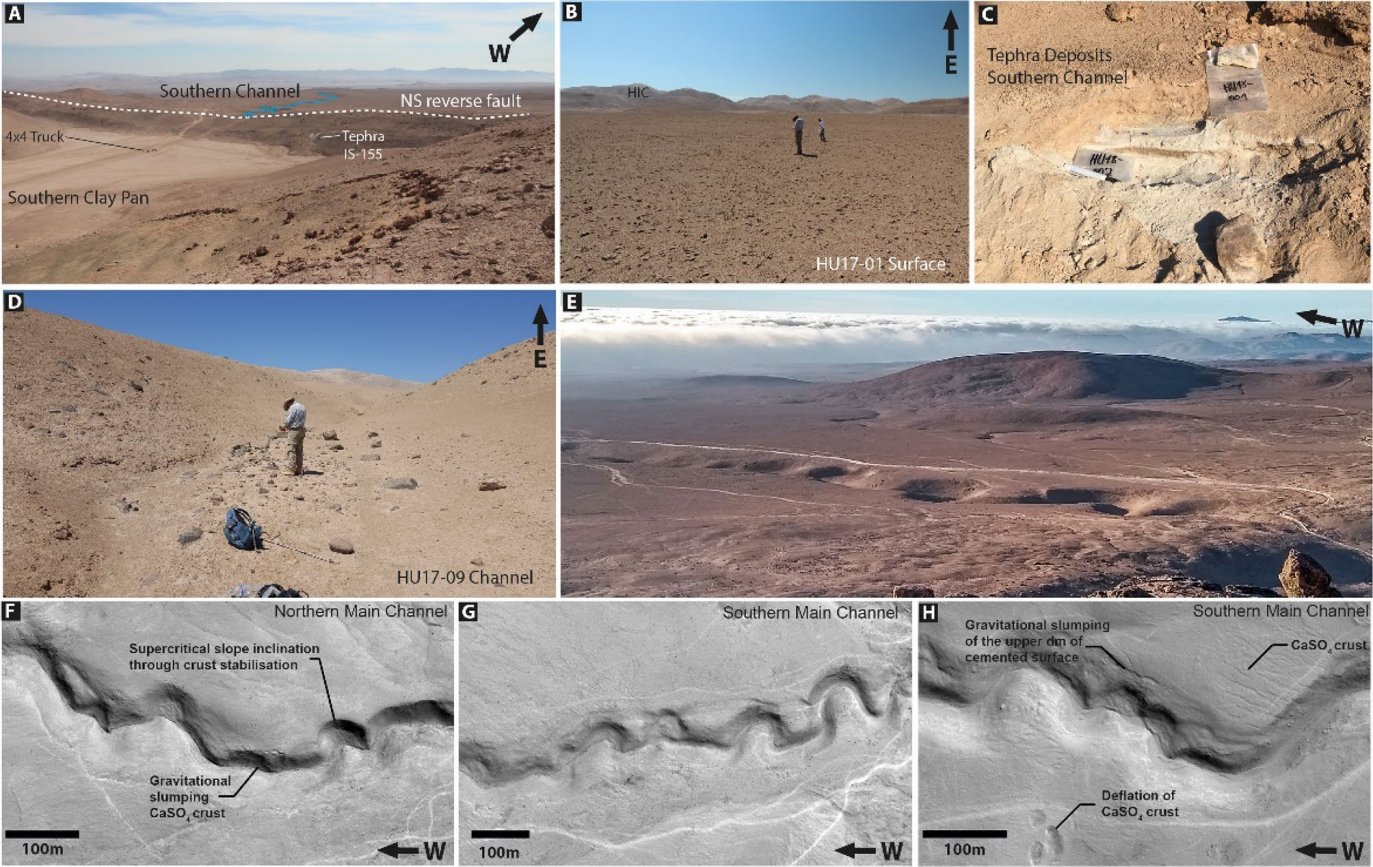
(**A**) View from an elevated area above the southernmost clay pan towards the west. The movement of the NS running reverse reactivated normal fault beheaded drainages to the west. Uplifted alluvium of the Altos de Hospicio gravels are outcropping along the fault scarp. A tephra layer (IS-155) located ~ 6 m below the crest of the tectonic fault scarp is dated to 22.9 ± 0.3 Ma^19^. (**B**) Fan-surface sampling for TCN exposure dating, here for HU17-01. Surfaces are generally relatively flat, areas with signs of localized recent fluvial erosion were avoided during sampling. The surface is covered by a thick CaSO_4_-rich soil. Desert pavement is not well evolved, due to the rarity of quartzes or other weathering resistant lithologies. (**C**) Channel sampling for TCN exposure dating, here for HU17-09. This channel incised ~ 5–6 m into unconsolidated alluvium of the Alto de Hospicio gravels. Samples were taken on the elevated sand bar within the channel to avoid sampling of pebbles rolled in from the channel’s slopes. (**D**) Two tephra beds were found in the southern channel that were covered a cm-thick CaSO_4_ -rich cover. Both tephra layers (HU18-001, HU18-002) lack lithic fragments, thus are presumably primary, not reworked. They are separated by a well-defined clastic layer, indicating that the tephra beds are from two separate eruptions. (**E**) View from a southern hilltop onto the southern main channel and alluvial fan surfaces. (**F**–**H**) Pléiades 1B panchromatic satellite imagery from the Huara site. (**F**) Northern main channel with gravitationally slumped blocks of the CaSO_4_ surface crust. Oversteepened channel slopes reflect the strong cohesion of the cemented surface cover. (**G**) Southern main channel with high sinuosity; steep channel slopes indicate no lateral incision into the channel. (**H**) Southern main channel with slumping features of the sulphate cover; adjacent areas show localized deflation of the CaSO_4_ cover.

Seemingly inactive highly sinuous to meandering stream channels are etched into the landscape in the eastern part of the northern Costal Cordillera, 30 km NE of Iquique in the Pampa de la Perdiz (Fig. 1). The E-W channels cut into the Alto Hospicio gravels (Fig. 1). These unconsolidated and partially salt-cemented upper Oligocene to Pliocene gravels, sands, silts and clays were deposited as sheet-, debris and locally sand and mud flows^19,20^. The absence of large outcrops made it difficult to study the internal composition and structure of the Alto Hospicio gravels. However, they are likely comparable to active alluvial fan system in the Precordillera and Central Depression to the East^21,22^. Alluvial fan surfaces dip moderately (mean ~ 4°) to the west and consist of erosional products of the elevated (max. ~ 1700 m) Huara Intrusive Complex (HIC, 140–136 Ma^19^) to the east. All alluvial fan surfaces are covered by a several decimetres to meter thick CaSO_4_-rich soil and crust sourced from atmospheric deposition^9,10,23^. Compressional tectonic forces caused a reverse reactivation of former normal faults during the Miocene along N–S and E–W running fault scarps^19^. Tectonic activity resulted in the beheading of the large depositional system to the west of the N-S-trending scarp. The fluvial channels were the final spilling pathways across the scarp, before they were cut from their source area (Figs. 1, 2). Fluvial channels are incised up to ~ 14 m into the crest of the thrust fault, indicating that initially incision kept pace with uplift. The tectonically truncated drainages terminate in endorheic basins filled by pan deposits to the east of the scarp^24^. All truncated channels crossing the scarp display distinct meanders with a sinuosity between 1.02 and 1.55 (see Supplementary Dataset), with highest values near the crest of the fault. The rims of the channel slopes on the outside of bends are commonly steeper than adjacent channel slopes due to induration by the regionally pervasive CaSO_4_ cover. The fluvial drainage system in the alluvial deposits can be best explained as confined sinuous to meandering channel. Although the fossil channels indicate a rather immature stage of evolution (i.e. floodplain did not form and the number of cut-offs is limited), they preserve original depositional fluvial features, such as mid-channel bars in straight sections (Fig. 2D). This is best observed at sites that are distal to the crest of the fault. Close to the crest, tectonic deformation and slumping of the channel slopes mostly obscure the original channel morphology. Occasionally upper CaSO_4_-rich layers protrude at channel slopes and cantilever failures as a consequence of the undermining of more erodible sediments beneath, indicating a significant difference in the strength of both materials. Fluvial channel bottoms are in some places covered by pristine tephra (Fig. 2D) which are covered by a friable CaSO_4_-rich cover. Channel slopes are close to the angle of repose of the Alto Hospicio gravels and/or their colluvium. No tributaries originate from the adjacent flat alluvial fan surfaces, indicating that runoff was dominantly sourced from the catchments in HIC (Fig. 1). The latter indicates that precipitation events during fluvial episodes, to the same extent affecting the entire area, did not modify fluvial surfaces on directly adjacent fan surfaces.

## Miocene surfaces and Plio-Pleistocene fluvial incision

**Figure 3:**
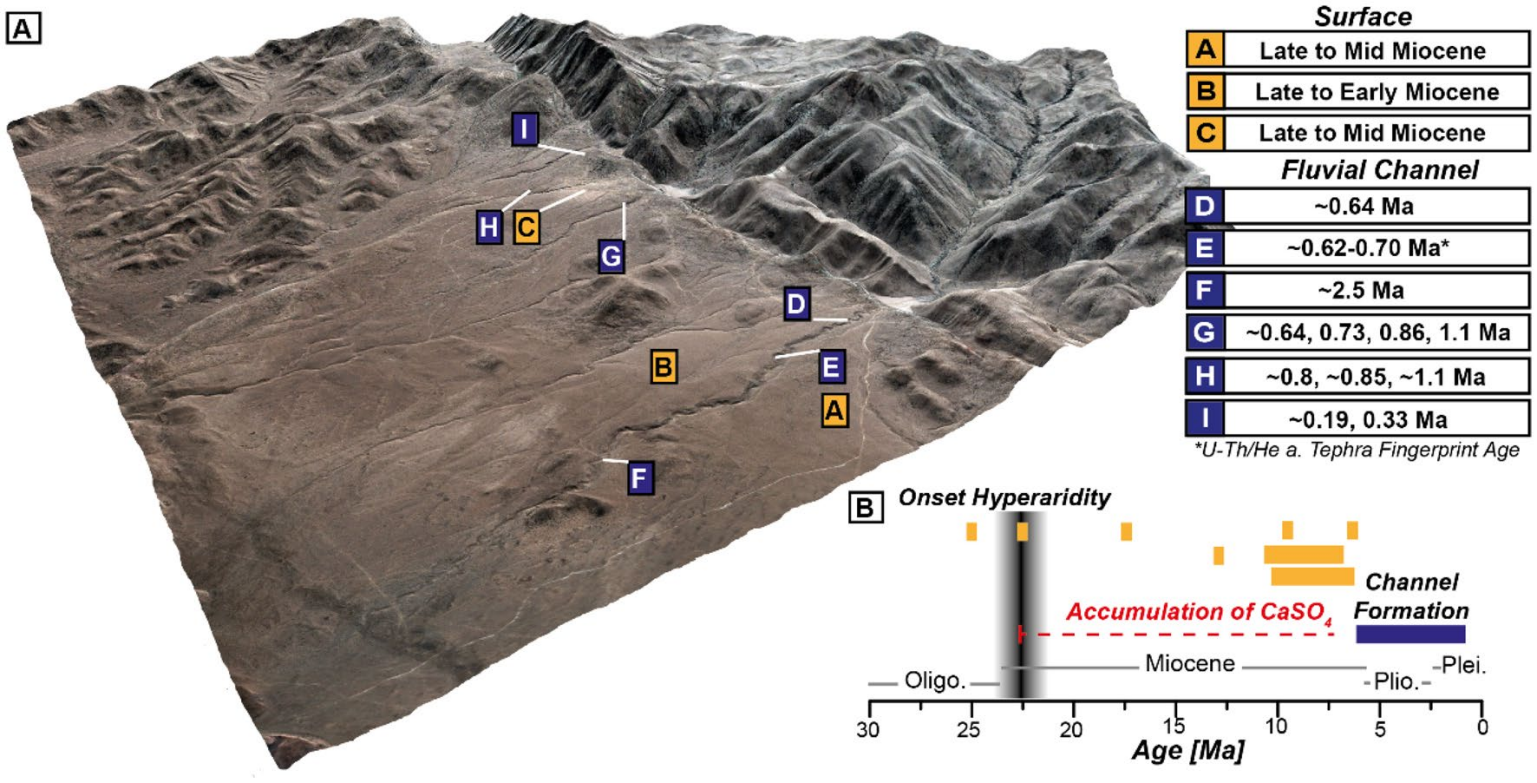
(**A**) 3D scene based on Pléiades 1B DEM and pansharped multispectral image created using ArcGIS Scene 10.5.1. Marked are sampling sites for dating, i.e., surfaces or channel beds. Compilation of age constrains for the landscape evolution of the sampling site, comprising ^21^Ne ages for ancient Miocene surfaces, ^10^Be ages for channel beds (multiple single clasts ages) and one age constraint based on geochemical fingerprinting of tephra layers. (**B**) Timeline for major landscape and climate evolution, comprising the early onset of hyperarid conditions at the Oligocene/Miocene boundary and the subsequent cessation of large deposition. Orange bars represent ^21^Ne exposure ages and/or their age range. Blue bar indicates time period of sinuous to meandering channel formation based on exposure ages. Detailed information about exposure ages and probability density plots are reported in the Supplementary Dataset.

Quartz clasts were sampled from alluvial fan surfaces and fluvial channel beds, as were two tephra layers from one channel bed, to determine the chronology of landscape evolution and constrain the environment under which sinuous to meandering channels evolved (Figs. 1, 2). The oldest ages (^21^Ne exposure ages 22–25 Ma) are from the alluvial fan surfaces to the east of the thrust fold (Fig. 3B), and point to long-term surface stability. This is in line with the notion that the region experienced predominantly (hyper-) arid conditions since the early Miocene^1,2,4,25^. Younger exposure age clusters are consistent with localized debris flow deposition onto the alluvial fans during the Mid-Miocene and a cessation of deposition after 5–6 Ma (Fig. 3), with the latter providing a maximum age-limit for the tectonic truncation of fan deposits from their source area. ^10^Be concentrations of fan-surface materials are saturated, confirming that exposure lasted longer than 4–5 Myr. Based on the ‘born at the surface model’ by Wells et al.^26^, an accretionary mantle of *‘soil-modified dust’* can build up beneath clasts previously exhumed to, or deposited onto, the surface. Therefore, the accumulation of atmospheric deposits took place after the initial exposure of the clasts. Any significant fluvial event will remove the clasts and erode the accretionary gypsic soil beneath. Long-term persistent aridity and the absence of any significant surface activity promoted the accumulation of CaSO_4_ and other clastic aeolian material since at least the deposition of the clasts and caused the preservation of a thick CaSO_4_ surface cover from atmospheric deposition^9,10,23^.

Tectonic ponding of the drainage to the east of the scarp presumably assisted in creating hydrologic conditions favourable for fluvial erosion (continuous flow, smoothing of peak discharges) further downstream before further tectonic uplift eventually beheaded these channels from their catchment source, creating fully closed endorheic basins. The tectonically created basins might acted as settling ponds for sediment transported during rare precipitation events, allowing bedload-free and sediment-poor water to overflow the tectonic barrier and subsequently cause incision. Rare and localised storm events in the Atacama Desert generated precipitation rates of 24–30 mm/h^27^. Based on disc infiltrometer measurements, Pfeiffer et al.^27^ estimated that precipitation has to exceed on average 78 mm/h for hillslopes to initiate infiltration/saturation-excess overland flow. Based on the recent occurrence of gypsic soils in the Atacama Desert mean annual precipitation probably did not exceed ~ 30 mm/year^9,23^. A sediment record from the southernmost endorheic clay pan confirms that fluvial deposition was restricted to these closed basins^24^. Besides removing the bulk of sediments from potential debris flows, the basins likely served as hydrologic buffers by flattening peak discharge and temporarily mimicking continuous flow during spilling events. Tectonic steepening of the alluvial fan slope near the crest may have promoted the initiation of, or further amplified^28^ high sinuosity^29,30^, ensuring that the flow remained confined.

Fluvial channels incised into the antecedent alluvial fan surface. This was at first capable of keeping pace with tectonic uplift but was later outpaced. ^10^Be and ^26^Al exposure ages from individual channel bed clasts and correlative dating of the channel tephra deposits within indicates that the drainages were terminally truncated at 640–800 ka in the southernmost catchment and ~ 185 ka in the northernmost catchment (Fig. 3). Based on the age-depth model of the southernmost clay pan climate record^24^ and the thickness of accumulated sediments it can be estimated that this pan received its current endorheic fill in the last 650–850 kyr. From the age of the alluvial fan surfaces (termination of fan-deposition) and channel deposits (termination of sediment transport, full cut-off), the sinuous to meandering channels incised sometime between the latest Miocene and late Quaternary. During this time, climatic conditions were arid to hyperarid on the Andean fore slope to the east of the study area^3,4^. Since the areas to the west of the Andean fore slope are generally drier (e.g.^1–4^), we also assume the study area was arid to hyperarid.

During this time, intermittent locally-sourced precipitation events must have been capable of incising the channels into the flat alluvial fans without modifying the surface. Vegetation cover is widely considered as one major prerequisite for adding strength to channel banks and slopes, so permitting high sinuosity of channels to develop in alluvial systems^31^. However, the absence of vegetation in the region requires another mechanism or process to enable the formation of these channel planforms by adding strength to channel banks and slopes.

## CaSO_4_-rich soil as landscape forming agent

### Preventing surface erosion

**Figure 4:**
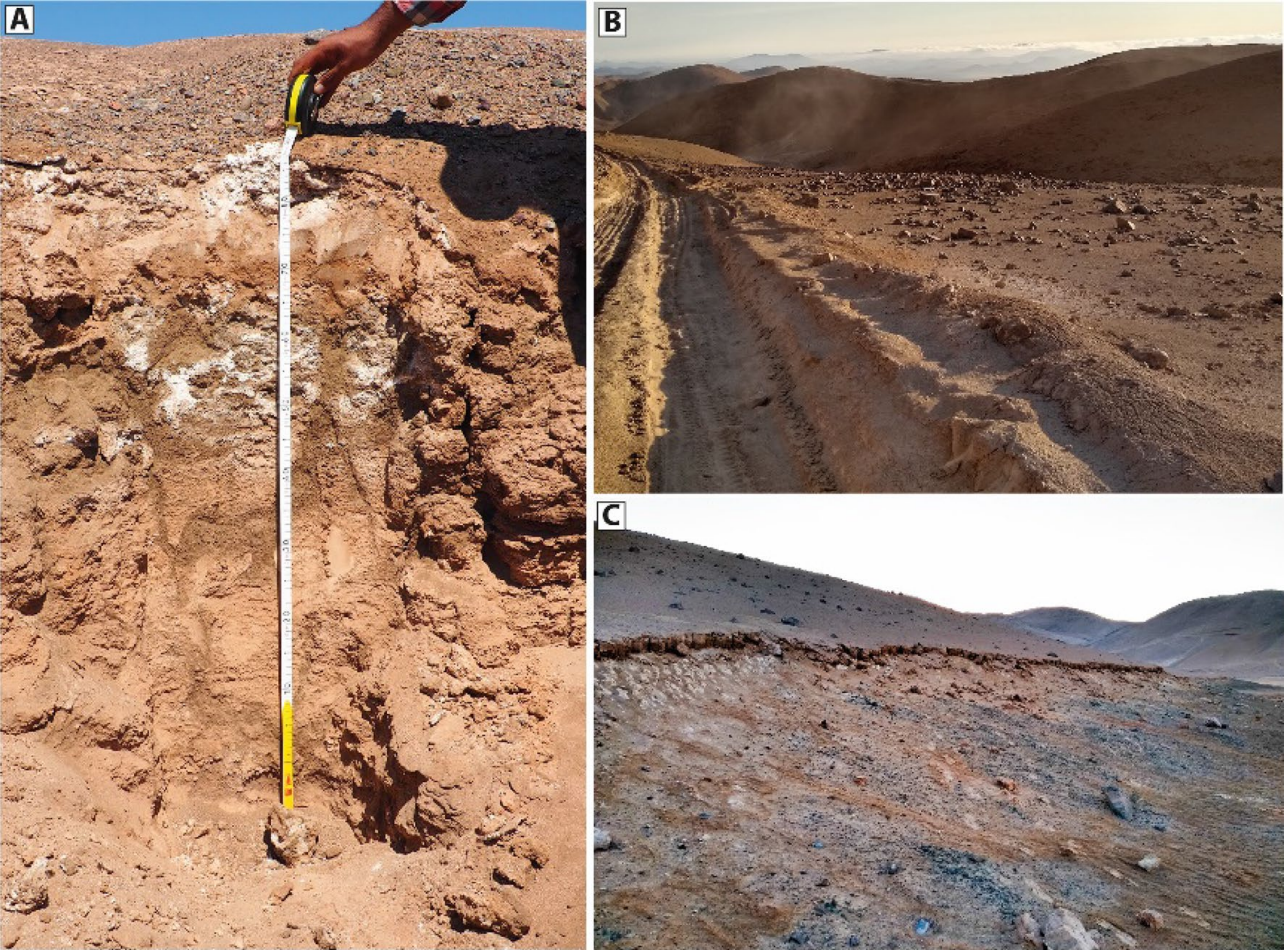
Typical occurrence of the ubiquitous CaSO_4_-rich soil in the study area. (**A**) Soil profile. (**B**) Powdery CaSO_4_ surface cover. (**C**) CaSO_4_-crust and subsurface cementation.

The old exposure ages of fan-surface clasts bear witness to hyperaridity and stability of the substrate since the Early Miocene. These conditions are a prerequisite for the accumulation and preservation of CaSO_4_-rich soils (Fig. 4). Long-term accumulation, preservation and modification of atmospheric CaSO_4_ deposition has led to cemented soils that cover much of the Atacama Desert^9–11,23,32–34^, that can be up to several meters thick^11,23,34^. Based on field observations, we ascertain a thickness of at least several decimetres to meters for our field site. Pedogenic processes which cause induration and cementation, include a combination of hydromorphic and illuvial processes^34,35^. These can be accentuated by diagenetic and surface exhumation^36^ and modified by the variable interaction of meteoric water or moisture; causing dissolution, redistribution and reprecipitation in the CaSO_4_-rich soil. The latter causes growth of CaSO_4_ and other soluble salts, leading to the cementation of the soil and underlying alluvial sediments (Fig. 4,^27,34–36^). These transformations are facilitated by moisture originating as rain, fog or dew. Dissolution and reprecipitation of CaSO_4_ phases by meteoric waters is verifiable through a poikilitic texture^23,34,36^. Surface dissolution and subsurface reprecipitation creates a thick, clast poor layer of almost purely CaSO_4_-rich material^11,23,34–36^. Anhydrite may be formed in the presence of highly saline solutions^10,32,37^ or due to dehydration of primary gypsum^37^. The uppermost soil layer, which is covered by a friable crust ^11^, usually remains powdery (‘*chusca*’ sensu Ericksen^11^), whereas concretions occur at depth (Fig. 4,^9,11^). Quantitative determination of the atmospheric deposition rate and soil inflation are difficult and rare from the Atacama Desert. Wang, et al.^10^ derived rates of ~ 35 cm/Ma soil accumulation based on meteoric ^10^Be data, which is in line with data from Placzek, et al.^38^. Soil accumulation rates using tephra deposits indicated also higher deposition rates of up 1 m/Ma^32^. The powdery layer promotes infiltration and prevents overland flow of any precipitation^27,39,40^. If the precipitation rate is below the infiltration capacity of the soil, overland flow will be prevented in favour of complete infiltration^15^. The infiltrated moisture drives dissolution and reprecipitation at depth, causing the induration of subsurface sediments^27,34,39,40^. Jordan et al.^40^ found indications that flat angle hillslopes and surfaces (< 7°, based on 90 m SRTM) experienced no measurable surface modifications, thus implying surficial covers of CaSO_4_ rich soils may hinder surface activity by absorbing rain. Precipitation falling the higher elevated catchments of the HIC were able to produce concentrated fluvial flows. Our study illustrates the role of this process in buffering the local erosional capacity of precipitation over millions of years, allowing the preservation of Miocene surfaces in direct proximity to Quaternary fluvial incisions.

### Adding strength: modulating fluvial incision

The development of sinuosity of a fluvial system, depends on several factors. Among them arguably the strength of parent rock or alluvium to resist fluvial erosion (flow resistance) dominates^41^. Strength can be provided or enhanced by sediment cohesiveness, bedrock strength or dense vegetation^41–44^. Riparian vegetation or a dense vegetation cover and bedrock strength can be clearly excluded here. Alternative mechanisms that may increase flow resistance in the absence of vegetation are: (1) permafrost^45^, (2) the cohesion of fine-grained sediments^18,44,46–48^, and (3) chemical cementation synchronous with channel flow^18,47,49^. These mechanisms may have promoted high sinuosity and meandering in arid to hyperarid environments in pre-vegetational streams of early Phanerozoic and Precambrian, and as potential analogues for sinuous to meandering channels on Mars^45–48,50–53^. None of these possibilities applies directly to this study. The alluvium of the Altos de Hospicio gravels might be the source of fine-grained sediments, however, strong prevailing winds presumably did not permit a significant deposition within the channels, which could be responsible for increasing strength and bank stability by cohesion. Fluvial systems with high sinuosity typically range between two extremes, bedrock/ice restricted channels with low amounts of alluvium involved, called incised or entrenched or channels fully developed in an alluvial setting (^54^^, and citations therein^), previously deposited in or near them (i.e. alluvial meander). Confined sinuous to meandering channels, as recognized in our study area, are therefore restricted in their lateral growth (forming/creating larger floodplains, cut-offs, etc.) by increased flow resistance of the sediment^41,54^. Hillslope failures by toe slope erosion and undercutting indicate that this increased flow resistance is only restricted to the upper part of the alluvium that is indurated by CaSO_4_. The observed channel forms therefore might be inherited, created during initial incision. A lack of discharge has probably hindered the migration of loops since the initial incision.

The case described here points to an additional mechanism that may promote higher sinuosity in a non-vegetated environment by adding strength to the sediments *prior to* incision in arid to hyperarid environments and dominance of atmospheric deposition. We posit that the regionally ubiquitous CaSO_4_-rich soils (Figs. 3, 4) that evolved from atmospheric deposition^9–11,32^ and subsequent pedogenic modification (e.g.^9,10,37^) provide the necessary strength to form high sinuosity of fluvial channels, restricting lateral erosion. This cementation of the upper sediments might mimic bedrock strength, ground-ice, or vegetation, causing higher sinuosity, although being confined, restricting/preventing the evolution to a fully meandering system in the first place. Carbonate as cement in this kind of hyperarid soil can be excluded as dominant ingredient, as ‘its formation is not chemically favoured in this hyperarid setting’^32^. CaSO_4_-wedges^9,11,55^ (‘sand wedges’^56^) and pressure regimes inside the soil due to dissolution and recrystallization may cause fracturing, similar to ignimbrites/bedrock, which promotes incision and channel formation, reflecting similar bedrock characteristics. Due to limited outcrop situation, we cannot fully exclude that cohesion or partly cementation of alluvial deposits additionally caused increased shear strength. However, the occurrence of a protruding CaSO_4_-rich soil and crust at the channels’ rims points CaSO_4_ cementation as the dominating factor for increased shear strength. We assume that the sinuous to meandering channel planforms were created during the initiation of fluvial incision into the strengthened upper meter or so of alluvium, and that subsequent fluvial flow was largely confined by the initial planform. Rapid channel incision into the CaSO_4_ cemented alluvium may have initially shaped the channel pattern with a subsequent inherited sinuosity. Fluvial periods presumably were not frequent or long enough to enable the evolution of floodplains, or the antecedent drainage was disconnected from the catchments by tectonic activity. Observed cantilever failures caused by the strength difference of the upper cemented alluvium and the weakly cemented and unconsolidated alluvium of the Altos de Hospicio gravels^19^ underlines the effect that pedogenic CaSO_4_ modification of sediments can have in the Atacama Desert. The stable pedogenically indurated substrate apparently provides the required sediment strength to maintain high sinuosity of channel forms. Capped duricrust are known to have a protective effect, resistant enough to preserve ancient landscapes^15,35^. The observation of sulphate-capped ridges from the Atacama Desert, resembling inverted fluvial stream channel indicate that sulphate cementation is able to increase the erosion resistance against fluvial and aeolian processes^57^. Studies of gypcrete in Egypt^36^ supports the protective nature of gypsum crusts against erosion and weathering, consolidating surface layers by cementation. The features observed here, such as bank failure/slumping by toe-slope erosion/cantilever failures of undercut banks, are equivalent to those commonly observed in environments with vegetated channel-banks^58^ (Fig. 2G,H).

The notion that antecedent encrustation/cementation, i.e., independent from and prior to channel flow, is a potential mechanism for surface induration and cementation that preserves landscape and adds strength to sediments, setting the foundation to enable high sinuosity in previously unconsolidated sediments in long-term predominantly hyperarid environments. Enduring atmospheric deposition and subsequent pedogenic cementation, i.e. the evolution of duricrusts, might have relevance to other arid to hyperarid climates and for the geomorphology of Mars, where similar channel forms are evident^45–48,50–53^. The Atacama Desert serves as a natural laboratory for potential surface processes on Mars due to the broadly similar climatic conditions and geochemical/mineralogical compositions^32,57,59^. Sulphates (albeit MgSO_4_^60^) are abundant on Martian surface^60–66^ and atmospheric deposition of sulphates from volcanic origin^67,68^ may give rise to surface encrustations that are similar to those found in the Atacama^9–11,27^. Mangold et al.^69^ have already indicated that Martian surfaces with polyhydrated sulphates in Candor Chasma are less eroded than the surfaces with monohydrated sulphates, which might be attributed to the formation of a duricrusts^69,70^.

The rates of and conditions for CaSO_4_ induration of sediments, and the build-up of CaSO_4_-rich soils, in the Atacama Desert will be the focus of future research. In particular, the conditions for cementation (state and volume of moisture involved, infiltration depth, and resulting cementation thickness) and their potential effects increasing sediment strength will be a focus. Experiments and models have to be conducted to further constrain this hypothesis and its effect on fluvial geomorphology in hyperarid settings, which is beyond the scope of this manuscript.

## Methods

We used geological field observations and a high-resolution Digital Elevation Model (DEM) of the study area, based on Pléiades 1B data (see Supplementary Dataset), to create swath profiles. Optical satellite imagery (Pléiades 1B Multispectral Image) was additionally used to identify geomorphologic features, such as the extent of alluvial fans and channels. Tephra layers in the southern main channel were geochemically characterized along with already dated and published tephra deposits from the local vicinity. Details about the lab procedure are outlined in the Supplementary Dataset. The chronology of the depositional systems (alluvial fans) and fluvial incision is reconstructed using exposure dating utilizing cosmogenic nuclides (^10^Be, ^26^Al, ^21^Ne). Exposure ages were determined for quartz clasts sampled during expeditions in 2014, 2015, and 2017. Clasts were sampled from flat fan surfaces (HU15-18, HU17-01, HU15-15), incised fluvial channels (HU14-05, HU15-16, HU17-09, HU15-14), and a channel terrace (HU17-04). Surface quartz clasts exhibit a reddish-brown desert varnish and are mostly angular. In cases of ‘kernsprung’, indicated by a localized cluster (usually < 2 m diameter) of quartz fragments, only one fragment per cluster was sampled and no other samples were collected within a 5 m radius. Quartz clasts from channels are partly rounded and have only a light desert varnish. The size of sampled clasts ranges between 2 and 5 cm. The abundance of quartz on the surfaces and channel beds is very low. Attempts to retrieve quartz from the subsurface for depth-profiling were impractical due to the CaSO_4_ indurated crust. To identify and correct for possible pre-exposure and exhumation we utilize the multiple clast approach^2,71,72^, using 5–7 clasts per sampling site. Further details to sampling sites and sampling details are given in the Supplementary Dataset. Quartz samples were crushed, sieved to retain the 250–710 µm fraction and etched several times in a dilute HF-HNO_3_ mixture^73^. Splits of the etched samples were used for ^10^Be, ^26^Al, and ^21^Ne analysis. ^10^Be and ^26^Al sample preparation and measurement followed the single stacked column approach detailed in^74^. Samples were measured at the CologneAMS^75^. Ne isotope analyses were conducted at SUERC (UK)^76^ and in Cologne (Germany)^77^. Further details about sampling preparation and measurement procedure are outlined in the Supplementary Dataset. Exposure ages were calculated using the LSDn scaling scheme of Lifton et al.^78^, as implemented in version 3 of ‘the online calculators formerly known as the CRONUS-Earth online calculators’ https://hess.ess.washington.edu/math/v3/v3_age_in.html described in^79^; see also Supplement. We assume a time integrated linear uplift of 40 m/Ma since 23 Ma, and report uplift-corrected ages accordingly (see Supplement).

## Supplementary Information

Supplementary Information.

## Data Availability

All data generated or analysed during this study are included in this published article (and its Supplementary Information files).

## References

[1] Dunai TJ, Lopez GAG, Juez-Larre J (2005). Oligocene-Miocene age of aridity in the Atacama Desert revealed by exposure dating of erosion-sensitive landforms. Geology.

[2] Ritter B (2018). Neogene fluvial landscape evolution in the hyperarid core of the Atacama Desert. Sci. Rep..

[3] Jordan TE, Kirk-Lawlor NE, Blanco PN, Rech JA, Cosentino NJ (2014). Landscape modification in response to repeated onset of hyperarid paleoclimate states since 14 Ma, Atacama Desert Chile. Geol. Soc. Am. Bull..

[4] Evenstar L (2017). Geomorphology on geologic timescales: Evolution of the late Cenozoic Pacific paleosurface in Northern Chile and Southern Peru. Earth-Sci. Rev..

[5] Houston J (2006). Variability of precipitation in the Atacama Desert: Its causes and hydrological impact. Int. J. Climatol..

[6] Garreaud RD, Molina A, Farias M (2010). Andean uplift, ocean cooling and Atacama hyperaridity: A climate modeling perspective. Earth Planet. Sci. Lett..

[7] Garreaud RD, Vuille M, Compagnucci R, Marengo J (2009). Present-day South American climate. Palaeogeogr. Palaeoclimatol. Palaeoecol..

[8] Houston J, Hartley AJ (2003). The Central Andean west-slope rainshadow and its potential contribution to the origin of hyper-aridity in the Atacama desert. Int. J. Climatol..

[9] Rech JA (2019). Massive middle Miocene gypsic paleosols in the Atacama Desert and the formation of the Central Andean rain-shadow. Earth Planet. Sci. Lett..

[10] Wang F (2015). Beryllium-10 concentrations in the hyper-arid soils in the Atacama Desert, Chile: Implications for arid soil formation rates and El Niño driven changes in Pliocene precipitation. Geochim. Cosmochim. Acta.

[11] Ericksen, G. E. *Geology and Origin of the Chilean Nitrate Deposits. Report No. 1188*, 37 (USGS, Washington, 1981).

[12] Binnie S (2020). The origins and implications of paleochannels in hyperarid, tectonically active regions: The northern Atacama Desert, Chile. Glob. Planet. Change.

[13] Tooth S (2000). Process, form and change in dryland rivers: A review of recent research. Earth Sci. Rev..

[14] Tooth S, Nanson GC (2000). Equilibrium and nonequilibrium conditions in dryland rivers. Phys. Geogr..

[15] Griffiths J, Fookes P, Goudie A, Stokes M (2012). Processes and landforms in deserts. Geol. Soc. Lond. Eng. Geol. Spec. Publ..

[16] Billi P, Demissie B, Nyssen J, Moges G, Fazzini M (2018). Meander hydromorphology of ephemeral streams: Similarities and differences with perennial rivers. Geomorphology.

[17] Santos MG (2019). Meandering rivers in modern desert basins: Implications for channel planform controls and prevegetation rivers. Sediment. Geol..

[18] Ielpi A (2019). Morphodynamics of meandering streams devoid of plant life: Amargosa River, Death Valley, California. GSA Bull..

[19] Vásquez, P. & Sepúlveda, F. Cartas Iquique y Pozo Almonte - Región de Tarapacá No. 161–163 Escala 1:100.000. *Carta Geológica de Chile Serie Geología Básica* (2013).

[20] Marquardt, R., Marinovic, S. & Muñoz, T. *Geología de las ciudades de Iquique y Alto Hospicio, región de Tarapacá, Escala* 1: 25.000. (2008).

[21] Morgan A (2014). Sedimentology and climatic environment of alluvial fans in the martian Saheki crater and a comparison with terrestrial fans in the Atacama Desert. Icarus.

[22] Kiefer E, Dorr MJ, Ibbeken H, Gotze HJ (1997). Gravity-based mass balance of an alluvial fan giant: The Arcas Fan, Pampa del Tamarugal, Northern Chile. Rev. Geol. Chile.

[23] Rech JA, Quade J, Hart WS (2003). Isotopic evidence for the source of Ca and S in soil gypsum, anhydrite and calcite in the Atacama Desert, Chile. Geochim. Cosmochim. Acta.

[24] Diederich JL (2020). A 68 ka precipitation record from the hyperarid core of the Atacama Desert in northern Chile. Glob. Planet. Change.

[25] Carizzo D, González G, Dunai TJ (2008). Constricción neógena en la Cordillera de la Costa, norte de Chile: Neotectónica y datación de superficies con 21Ne cosmogénico. Rev. Geol. Chile.

[26] Wells SG, McFadden LD, Poths J, Olinger CT (1995). Cosmogenic ^3^He surface exposure dating of stone pavements. Geology.

[27] Pfeiffer M (2021). Century scale rainfall in the absolute Atacama Desert: Landscape response and implications for past and future rainfall. Quatern. Sci. Rev..

[28] Dente E, Lensky NG, Morin E, Enzel Y (2021). From straight to deeply incised meandering channels: Slope impact on sinuosity of confined streams. Earth Surf. Process. Landf..

[29] Hooke, J. M. *River Meandering*. (2020).

[30] Schumm SA (2007). River Variability and Complexity.

[31] Ielpi A, Lapôtre MG, Gibling MR, Boyce CK (2022). The impact of vegetation on meandering rivers. Nat. Rev. Earth Environ..

[32] Ewing SA (2006). A threshold in soil formation at Earth's arid-hyperarid transition. Geochim. Cosmochim. Acta.

[33] Rech JA, Currie BS, Michalski G, Cowan AM (2006). Neogene climate change and uplift in the Atacama Desert, Chile. Geology.

[34] Hartley AJ, May G (1998). Miocene gypcretes from the Calama Basin Northern Chile. Sedimentology.

[35] Watson A (1988). Desert gypsum crusts as plaeoenvironmental indicators: A micropetrographic study of crusts from southern Tunisia and the central Namib Desert. J. Arid Environ..

[36] Aref MA (2003). Classification and depositional environments of Quaternary pedogenic gypsum crusts (gypcrete) from east of the Fayum Depression, Egypt. Sediment. Geol..

[37] Voigt C, Klipsch S, Herwartz D, Chong G, Staubwasser M (2020). The spatial distribution of soluble salts in the surface soil of the Atacama Desert and their relationship to hyperaridity. Glob. Planet. Change.

[38] Placzek C, Quade J, Rech JA, Patchett P, de Arce CP (2009). Geochemistry, chronology and stratigraphy of Neogene tuffs of the Central Andean region. Quat. Geochronol..

[39] May SM (2020). Origin and timing of past hillslope activity in the hyper-arid core of the Atacama Desert-The formation of fine sediment lobes along the Chuculay Fault System, Northern Chile. Glob. Planet. Change.

[40] Jordan, T. *et al.**XIV Congreso Geologico Chileno (La Serena).*

[41] Lazarus ED, Constantine JA (2013). Generic theory for channel sinuosity. Proc. Natl. Acad. Sci. USA.

[42] Tal M, Paola C (2007). Dynamic single-thread channels maintained by the interaction of flow and vegetation. Geology.

[43] Braudrick CA, Dietrich WE, Leverich GT, Sklar LS (2009). Experimental evidence for the conditions necessary to sustain meandering in coarse-bedded rivers. Proc. Natl. Acad. Sci. USA.

[44] Howard AD (2009). How to make a meandering river. Proc. Natl. Acad. Sci. USA.

[45] Fairén, A., Davies, N. S. & Squyres, S. *44th Lunar and Planetary Science Conference, Abstract.*

[46] Lapôtre MG, Ielpi A, Lamb MP, Williams RM, Knoll AH (2019). Model for the formation of single-thread rivers in barren landscapes and implications for pre-Silurian and martian fluvial deposits. J. Geophys. Res..

[47] Matsubara Y (2015). River meandering on Earth and Mars: A comparative study of Aeolis Dorsa meanders, Mars and possible terrestrial analogs of the Usuktuk River, AK, and the Quinn River, NV. Geomorphology.

[48] McMahon WJ, Davies NS (2018). The shortage of geological evidence for pre-vegetation meandering rivers. Fluvial Meanders Sediment. Prod. Rock Rec..

[49] Gibling MR, Rust BR (1990). Ribbon sandstones in the Pennsylvanian Waddens Cove Formation, Sydney Basin, Atlantic Canada: The influence of siliceous duricrusts on channel-body geometry. Sedimentology.

[50] Kereszturi, Á. *Fluvial Geomorphology of Mars: Background to Separate Biogenic and Abiogenic Effects and to Identify Climate Change Related Features*. (2015).

[51] Lapotre, M. G. A. & Ielpi, A. *AGU Fall Meeting Abstracts.*

[52] Lapôtre MG, Ielpi A (2020). The pace of fluvial meanders on Mars and implications for the western delta deposits of Jezero crater. AGU Adv..

[53] Ielpi A, Lapôtre MG (2019). Barren meandering streams in the modern Toiyabe Basin of Nevada, USA, and their relevance to the study of the pre-vegetation rock record. J. Sediment. Res..

[54] Allen J (1982). Free meandering channels and lateral deposits. Sediment. Struct..

[55] Zinelabedin A, Riedesel S, Reimann T, Ritter B, Dunai TJ (2022). Testing the potential of using coarse-grain feldspars for post-IR IRSL dating of calcium sulphate-wedge growth in the Atacama Desert. Quat. Geochronol..

[56] Sager C, Airo A, Arens FL, Schulze-Makuch D (2021). New type of sand wedge polygons in the salt cemented soils of the hyper-arid Atacama Desert. Geomorphology.

[57] Williams RM (2021). Inverted channel variations identified on a distal portion of a bajada in the central Atacama Desert, Chile. Geomorphology.

[58] Merritt DM (2020). Reciprocal Relations between Riparian Vegetation Fluvial Landforms and Channel Processes.

[59] Azua-Bustos A, González-Silva C, Fairén AG (2022). The Atacama Desert in Northern Chile as an analog model of Mars. Front. Astron. Space Sci..

[60] Ehlmann BL, Edwards CS (2014). Mineralogy of the Martian surface. Annu. Rev. Earth Planet. Sci..

[61] Bibring J-P (2006). Global mineralogical and aqueous Mars history derived from OMEGA/Mars Express data. Science.

[62] Christensen, M. O., Hamilton, V., Edwards, C., Wray, J. & Anderson, F. S. *Aqueous Mineral Deposits in an Ancient, Channeled, Equatorial Terrain*. PR (2008).

[63] Osterloo M (2008). Chloride-bearing materials in the southern highlands of Mars. Science.

[64] Mustard JF (2008). Hydrated silicate minerals on Mars observed by the Mars Reconnaissance Orbiter CRISM instrument. Nature.

[65] Poulet F (2005). Phyllosilicates on Mars and implications for early Martian climate. Nature.

[66] Sharp RP, Malin MC (1984). Surface geology from Viking landers on Mars: A second look. Geol. Soc. Am. Bull..

[67] Settle M (1979). Formation and deposition of volcanic sulfate aerosols on Mars. J. Geophys. Res..

[68] Franz HB, King PL, Gaillard F (2019). Volatiles in the Martian Crust.

[69] Mangold N (2008). Spectral and geological study of the sulfate-rich region of West Candor Chasma, Mars. Icarus.

[70] Robertson K, Bish D (2013). Constraints on the distribution of CaSO4· nH2O phases on Mars and implications for their contribution to the hydrological cycle. Icarus.

[71] Binnie A (2016). Accelerated late quaternary uplift revealed by 10 Be exposure dating of marine terraces, Mejillones Peninsula, northern Chile. Quat. Geochronol..

[72] Farbod Y (2016). Spatial variations in late Quaternary slip rates along the Doruneh Fault System (Central Iran). Tectonics.

[73] Kohl C, Nishiizumi K (1992). Chemical isolation of quartz for measurement of in-situ-produced cosmogenic nuclides. Geochim. Cosmochim. Acta.

[74] Binnie SA (2015). Separation of Be and Al for AMS using single-step column chromatography. Nucl. Instrum. Methods Phys. Res. Sect. B.

[75] Dewald A (2013). CologneAMS, a dedicated center for accelerator mass spectrometry in Germany. Nucle. Instrum. Methods Phys. Res. Sect. B.

[76] Codilean AT (2008). Single-grain cosmogenic ^21^Ne concentrations in fluvial sediments reveal spatially variable erosion rates. Geology.

[77] Ritter B, Vogt A, Dunai TJ (2021). Technical Note: Noble gas extraction procedure and performance of the Cologne Helix MC Plus multi-collector noble gas mass spectrometer for cosmogenic neon isotope analysis. Geochronology.

[78] Lifton N, Sato T, Dunai TJ (2014). Scaling in situ cosmogenic nuclide production rates using analytical approximations to atmospheric cosmic-ray fluxes. Earth Planet. Sci. Lett..

[79] Balco G, Stone JO, Lifton NA, Dunai TJ (2008). A complete and easily accessible means of calculating surface exposure ages or erosion rates from (10)Be and (26)Al measurements. Quat. Geochronol..

