# Supplementary material for: Impact of CaSO4-rich soil on Miocene surface preservation and Quaternary sinuous to meandering channel forms in the hyperarid Atacama Desert
Source: Sci Rep. 2022 Oct 26;12:17951. doi: 10.1038/s41598-022-22787-9 (PMC9606260; doi:10.1038/s41598-022-22787-9)
Supplement: Supplementary file 1 — Supplementary Information. [file 41598_2022_22787_MOESM1_ESM.pdf]

Supplementary Information S1

Impact of $CaSO_4$-rich soil on Miocene surface preservation and Quaternary sinuous to meandering channel forms in the hyperarid Atacama Desert

Benedikt Ritter [1], Julia L. Diederich-Leicher [1], Steven A. Binnie [1], Finlay M. Stuart [2], Volker Wennrich [1], Andreas Bolten [3], Tibor J. Dunai [1]

1 Institute of Geology & Mineralogy, University of Cologne, Germany

2 Isotope Geosciences Unit, Scottish Universities Environmental Research Centre, East Kilbride, UK

3 Institute of Geography, University of Cologne, Germany

1. Regional Setting

The study area is situated in the Coastal Cordillera (Fig. S1), an eroded Jurassic magmatic arc. During its activity in the Jurassic and early Cretaceous, extensive volcanic sequences formed and several plutons intruded [1,2]. The Huara Intrusive Complex (HIC) is located to the east of the studied fluvial system. Intercalated marine sedimentary rocks, formed as a late Jurassic to early Cretaceous basin fill, and are now exposed, particularly in the eastern part of the Coastal Cordillera [2]. A large hiatus occurred between the early Cretaceous and the Oligocene-Miocene (maximum age), the latter indicated by the deposition of the Alto Hospicio gravels (*Gravas de Alto Hospicio*), their composition and shape reflect only local erosional products [2]. Deposition took place in the pre-existing relief and structural depressions (hemigraben generated by extensional tectonics), generating the low relief Tarapacá pediplain, e.g. [3].

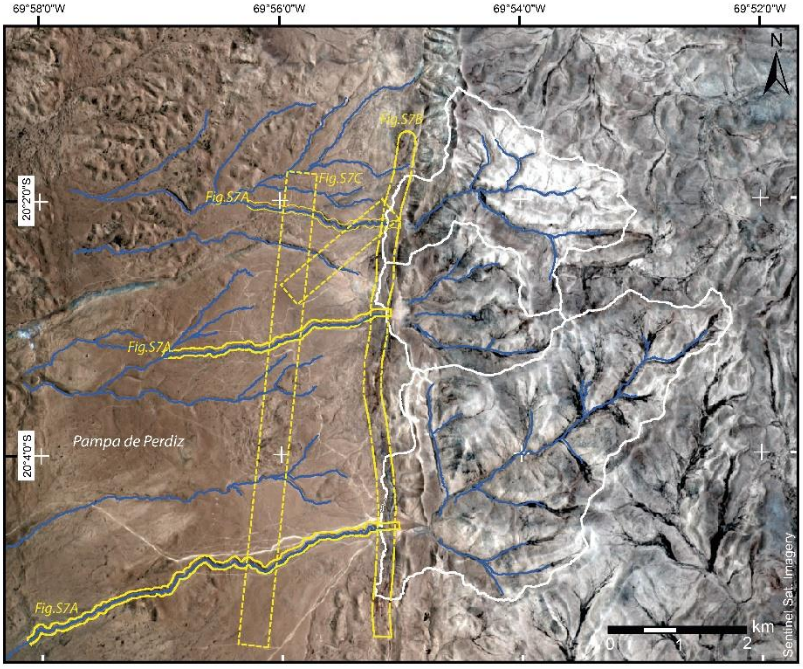

Fig. S1: Overview map of the study area based on Sentinel 1B satellite imagery (created using ArcGIS Pro 2.8.1 - pro.arcgis.com - and Adobe Illustrator 2022 -adobe.com). Blue lines represent fluvial channels. White encircled areas represent delineate endorheic catchments. Yellow areas mark swath profiles see Fig. S7.

2. Sampling Site and Material

Several tephra beds occur throughout most of the catchments of the HIC with varying thickness, thickest in topographic lows, such as channels. Two newly identified tephra layers were found on top of each other in the channel bed of the southernmost main channel (HU18-001, HU18-002, Fig. 3D), distinctly separated by a thin dust layer. Both tephra layers are not consolidated nor cemented, consist of pristine glass without significant xenolithic fragments, and thus can be considered as primary deposits.

Additionally, four tephra samples from the HIC and its vicinity [2] were re-sampled in order to establish geochemistry-based correlations that enable the extrapolation of ages. Three of these tephra beds have ages in the range of 2.06 to 0.62 Ma (biotite $^{40}Ar/^{39}Ar$ [2]). One tephra layer (IS-155) intercalated in alluvial gravels of the Alto de Hospicio Gravels and outcropping in the uplifted tectonic dam in the southernmost clay pan yields an age of 22.9±0.3Ma (Ar/Ar on biotite[2]).

Alluvial fan surface quartz samples for cosmogenic nuclide exposure dating were collected from three fan surfaces (HU15-018, HU17-001, HU15-015, sampling area ~100m^2) on the Pampa de la Perdiz west of the HIC. No signs of recent fluvial activity were observed. The surfaces dip towards the west (mean ~4°) and are covered by calcium-sulphate-rich soils. Surfaces exhibit only a sparse desert pavement. Consulting the 'born at the surface model' of Wells, et al. [4], rocks of a desert

pavement remain at the surface on an accretionary mantle of soil-modified atmospheric dust and are continuously raised from the original/primary sediment surface. Wang, et al. [5] suggests that this process is operational in hyper-arid soils of the Atacama Desert, allowing the continuous exposure of clasts on the surface of alluvial fans. Clasts will be kept at the surface, even though modifications of the soil by deflation or moderate fluvial erosion occur. Quartz clasts are predominantly vein-quartz, which is a trace constituent of the outcropping HIC. They are the dominant clasts, fine-grained and/or polymineralic clasts were presumably removed by weathering and erosion. The majority of sampled quartz clasts have a dark reddish desert varnish, indicative for long-lasting surface exposure. Most of the surface clasts have angular shapes, though some retain fluvial rounding. Clusters of quartz clasts were assumed to derive from 'kernsprung' (insolation weathering), hence, only one clast was sampled in a radius of 2 m to avoid distortion of the age distribution of single-clast ages. The small spatial dispersion of kernsprung-fragments (<2m) points to long-term surface stability and only limited diffusive transport.

Four channel beds (HU14-05, HU15-016, HU17-09, HU17-14) were sampled for cosmogenic nuclide exposure dating, aiming to date the onset of inactivity due to abandonment. Fluvial channels formed single tortuous structures with sinuosity between 1.02-1.55 [6], especially channels with former, but still visible, connections to adjacent clay pans. Channels have deeply incised into uplifted alluvial strata along the NS running reverse reactivated normal fault. Channels incised on average approximately 9-15.5 m into unconsolidated alluvium (maximum incision between 18-40 m, the latter affected by outcropping bedrock – satellite bodies from the HIC). Depth of incision and channel hillslope steepness decreases towards the west. Channel profiles (Fig. S7) reveal steeper channel gradients in the northern channels as compared to the southern ones. The two northernmost channels traverse a NE-SW running reverse fault, which caused additional uplift and tilting of the southern areas. Channel slopes are mostly at the angle of repose and are covered in loose debris. Channel beds are filled with sand and fine gravel, commonly covered by a $CaSO_4$-rich dust-layer, the latter fixated by a friable crust. Larger pebbles and boulders are only observed in close proximity to the channel-clay pan transition, sources are the tectonic fragmentation of bedrock near the scarp or/and the proximal rocks of the HIC. Sampled clasts were collected from the crest of sediment bars within the channels to avoid material that may have rolled in from the channel slopes. Channel bed clasts exhibit only a slight desert vanish and are mostly angular in shape.

In addition to the existing channel bed samples, one gravel terrace within the southern main channel was sampled (HU17-004). The terrace is situated at the southern slope/bank of the meander, approximately ~5,000 m^2 in areal extent (~1071 m) and up to ~6 m above the recent channel bed (~1065 m) and ~8 m below the adjacent alluvial fan surface (~1079 m). The terrace was presumably built when the channel incised further than ~8 m below the fan surface before it

was subsequently abandoned. The top of the terrace is relatively flat and covered by boulders. Few quartz clasts could be found, mostly angular with minor desert varnish.

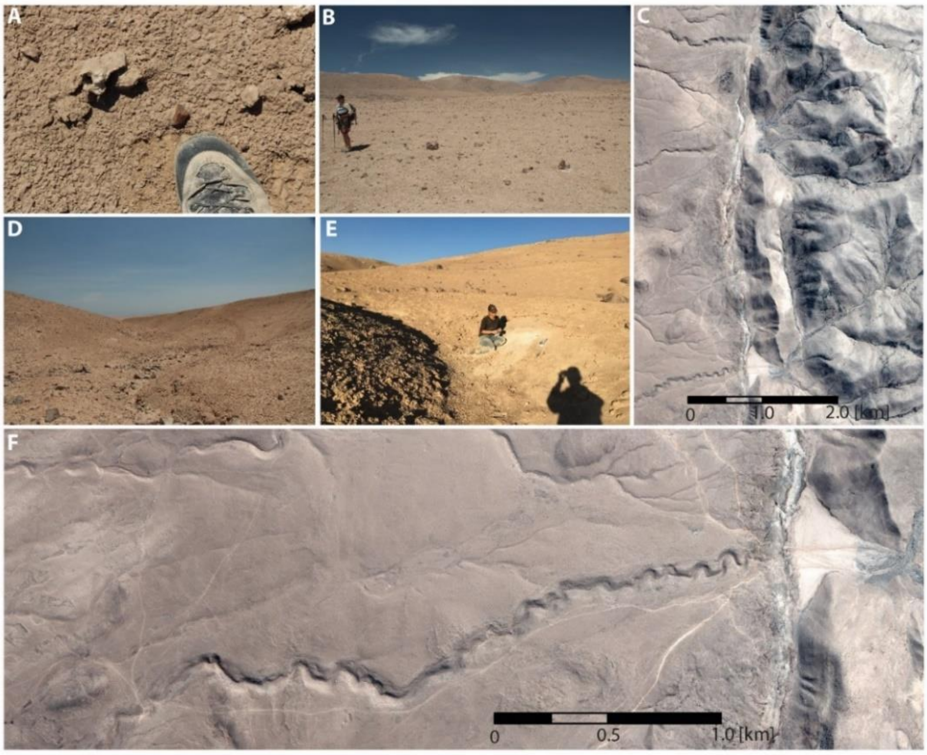

Fig. S2: A.) Surface of sampling site HU17-001. Quartz clasts are rare on the surface and have a brownish desert vanish. Additionally, the photograph illustrates the calcium-sulphate-rich surface cover, including extensive crusts and smaller concretions. B.) View from sample site HU15-15 towards the HIC. C.) Pléiades 1B pansharped multi-spectral image of the NS running reverse fault system. Visible are three clay pans to the east of the uplifted dam, which act as main depocenters for erosional products from the HIC catchments. D.) Photograph towards the west from within the northernmost sampled fluvial channel (HU15-14). E.) Southern fluvial channel indicating the sampling site of the two important tephra deposits (HU18-001, 002). F.) Pléiades 1B pansharped multi-spectral image of the entire southern fluvial channel. Higher sinuosity is visible in the upper part close to the uplifted dam.

Table S1: Sampling site details.

Sample ID	Type	Latitude	Longitude	Elevation [m]
HU14-05	TCN Channel	S 20° 4'37.21"	W 69°55'21.80"	1222
HU15-14	TCN Channel	S 20°02'11.80''	W 69°55'13.00''	1329
HU15-15	TCN Surface	S 20°02'34.40''	W 69°55'26.50''	1285
HU15-16	TCN Channel	S 20°02'59.20''	W 69°55'25.80''	1289
HU15-18	TCN Surface	S 20°05'26.50''	W 69°56'01.20''	1111
HU17-01	TCN Surface	S 20° 4'29.55"	W 69°56'2.36"	1142
HU17-04	TCN Terrace	S 20° 4'57.90"	W 69°56'51.42"	1052
HU17-09	TCN Channel	S 20° 2'24.19"	W 69°55'43.58"	1243
HU18-001	Tephra	S 20° 4'45.42"	W 69°55'49.61"	1166
HU18-002	Tephra	S 20° 4'45.42"	W 69°55'49.61"	1166

3. Methods

Digital Elevation Model (DEM)

The Shuttle Radar Topography Mission (SRTM) DEM data was used for larger scale geomorphological analysis of the entire HIC. High resolution stereo satellite image sets obtained from Pléiades 1B system were used for swath profiles of meander systems and surfaces of the Huara study site. Image recording was on 17.04.2014 with a ground resolution of 0.5 m in the panchromatic band. ExelisVIS ENVI 5.1 was used to derive a surface model with the DEM Extraction module. Tie point determination between the two stereo images was conducted manually. Post-processing tools, including filter methods to reduce spikes (ENVI DEM Editing tools), were applied. The final model was resampled to a resolution of 2 m. A pansharped image was produced using ExelisVIS ENVI 5.1. Optical satellite imagery (Pléiades 1B Multispectral Image and Sentinel 1B) was used to identify landscape features, such as alluvial fan extensions and channels. Further GIS analysis used ArcGIS® 10.5.1 (ESRI http://www.esri.com), such as watershed delineation and swath profile creation.

Tephrochronology - Geochemical Fingerprinting

Tephra deposits were washed in de-ionized water, sieved >32 μm and <125 μm, and subsequently treated by magnetic separation (FRANTZ) to remove clay and salt contamination and to purify glass shards. Glass concentrates were imbedded in epoxy, polished, and subsequently carbon coated prior to electro microprobe analysis (EPMA). Element compositions of sample glass shards and secondary reference material ATHO-G, [7,]Lipari ID3506 obsidian, [8] are analysed by electron probe microanalysis using a JEOL JXA-8900RL at the University of Cologne. The JEOL JXA-8900RL is equipped with a five-wavelength dispersive spectrometer, which was set to 12 keV accelerating voltage, 6 nA beam current, and 5 μm beam diameter, respectively. Element compositions were normalized to 100% anhydrous and volatile-free. Detailed information about counting times, reference materials etc. are provided in the supplementary datafile.

Cosmogenic nuclide exposure dating

Samples were ground, sieved to 250-710 μm and subsequently purified by sequential HF-leaching [9]. Purified quartz separates were investigated under a microscope. Those with a high abundance of visible fluid inclusions and/or fragments resembling chalcedony were further etched or excluded from ^{21}Ne analysis. Inductively coupled plasma-optical emission spectrometry (ICP-OES) was used to verify the purity of the quartz before dissolution for ^{10}Be and ^{26}Al. Splits of the etched material were used for ^{10}Be, ^{26}Al and ^{21}Ne analysis.

Etched quartz samples were dissolved following spiking with certified Be (Scharlab, 1000 mg/l) and Al (Scharlab, 1000 mg/l) standard solutions. AMS (accelerator mass spectrometry) target preparation followed the stacked column approach from [10], co-precipitating Al and Be hydroxides

with Ag following [11]. Chemical blanks were prepared alongside the samples. $^{10}Be/^{9}Be$ and $^{26}/^{27}Al$ values were measured on CologneAMS [12]. $^{10}Be/^{9}Be$ ratios were normalized to the ICN standard dilution series of [13] and $^{26}Al/^{27}Al$ ratios were normalized to the ^{26}Al AMS standards provided by [14]. For $^{26}Al/^{27}Al$ samples, the stable Al contents of the dissolved, spiked samples were determined using ICP-OES and standard addition (4 aliquots) in tandem with quality control measurements of NIST SRM165a. Blank corrected concentrations of ^{10}Be and ^{26}Al were derived following the procedure outlined in Binnie, et al. [15]. Concentration uncertainties include propagated uncertainties in the AMS ratios together with the estimated standard deviation of ^{9}Be, or ^{27}Al, mass that the samples contained after spiking. To gain knowledge about potential complex exposure histories, i.e. temporal burial, $^{26}Al/^{10}Be$ ratios vs respective ^{10}Be concentrations were plotted using "The online exposure age calculator formerly known as the CRONUS-Earth online exposure age calculator." Version 3, http://hess.ess.washington.edu/math/v3/v3_age_in.html; [16]. These two-isotope plots were calculated using the LSDn scaling scheme [17]. Uncertainties are displayed as 1σ.

For cosmogenic ^{21}Ne, cleaned and purified samples were packed into aluminium foil cups. The samples were measured with a noble-gas mass spectrometer at Scottish Universities Environmental Research Centre (SUERC) applying the standard procedure, including correction for isobaric interferences at mass 20,21, and 22 [18-20]. Additional samples were measured at the University of Cologne Institute of Geology and Mineralogy applying the standard procedure outlined in [21].

Ages were derived using the 'nuclide dependent scaling' after Lifton, et al. [17], calculated with "The online exposure age calculator formerly known as the CRONUS-Earth online exposure age calculator." Version 3, http://hess.ess.washington.edu/math/v3/v3_age_in.html; [16]. Reference production rates were determined using the CRONUS-Earth calibration dataset [22]. Topographic shielding was representative measured at one meander sampling site. We applied a time-integrated inverse modelling of cosmogenic nuclide production rates by subsequent modelled subsidence to paleo elevations. We used a linear uplift model of 40 m/Ma [23,24] with uplift commencing at ~23 Ma (assuming that uplift of the alluvial fans started from a vertical position). To apply the uplift correction using LSDn scaling as implemented the CRONUS-Earth online calculator (V.3), we calculated the uplift-dependant change by using an erosion rate (assuming the reduction of the mass air above the sample by uplift is mathematically equivalent to erosion). The density of air decreases with altitude (rising from sea-level to ~1200 m, our mean topographic elevation of the area) the density of air is reduced by ~11% (U.S. Standard Atmosphere 1976, NASA-TM-X-74335). For the purpose of calculating the effect of atmospheric mass removal by uplift we utilize the mean density of air at 'elevation sampling site/2' (1.15-1.17 kg/m^3, NASA-TM-X-74335). The $\rho_{air\ elevation/2}/\rho_{Qtz}$ multiplied by the uplift rate provides the 'erosion

rate' (in our case 1.74-1.76 $x*10^{-6}$ cm/yr) for input into the online calculator. For samples with concentrations implying an age >23 Ma, we calculated the excess of ^{21}Ne concentration from a sample near this age (HU17-01F, 22.7 Ma) and applied an averaged ^{21}Ne production rate for the initial sampling site elevation (for HU17-01 222 m a.s.l.) to calculate the excess age. The latter is added to the 'wiggle' age of HU17-01F. Internal uncertainty reflects the analytical uncertainty, additionally we assigned a 10% external uncertainty to our ages to account for the lack of detailed data available to constrain the paleo magnetic field strength over the last several million years as well as uplift rate uncertainties.

4. Results

Tephrochronology

Geochemical Fingerprinting

Geochemical fingerprinting of individual glass shards yielded a rhyolithic composition for all investigated tephra samples. The glass major and minor element composition of samples HU18-001, and HU18-002 from the southern channel bed reveal distinct clusters, proving the primary deposition of the tephra beds and pointing to separate volcanic sources and/or eruptions. Bioxide plots (Fig. S3) indicate a clear overlap of the HU18-002 cluster with clusters of two tephra samples IV-190 and CMI13.3 from the HIC and its vicinity, both dated by $^{40}Ar/^{39}Ar$ on biotite to 0.62±0.04 Ma and 0.7±0.1 Ma, respectively [2]. All three tephra samples build a specific distinct cluster, with lower SiO_2, but higher TiO and FeO contents (Fig. S3). Sample HU18-001, the uppermost channel bed tephra, does not yield a conclusive match with any of the reference tephra samples. However, HU18-001 show overlapping clusters with sample IV-189 (=HU18-008; 2.06±0.12 Ma) in most biplots, but with higher Cl contents (Fig. S3). This could indicate a common volcanic source of both tephra beds, but different eruption events. Geochemical data is available as supplementary data excel sheet.

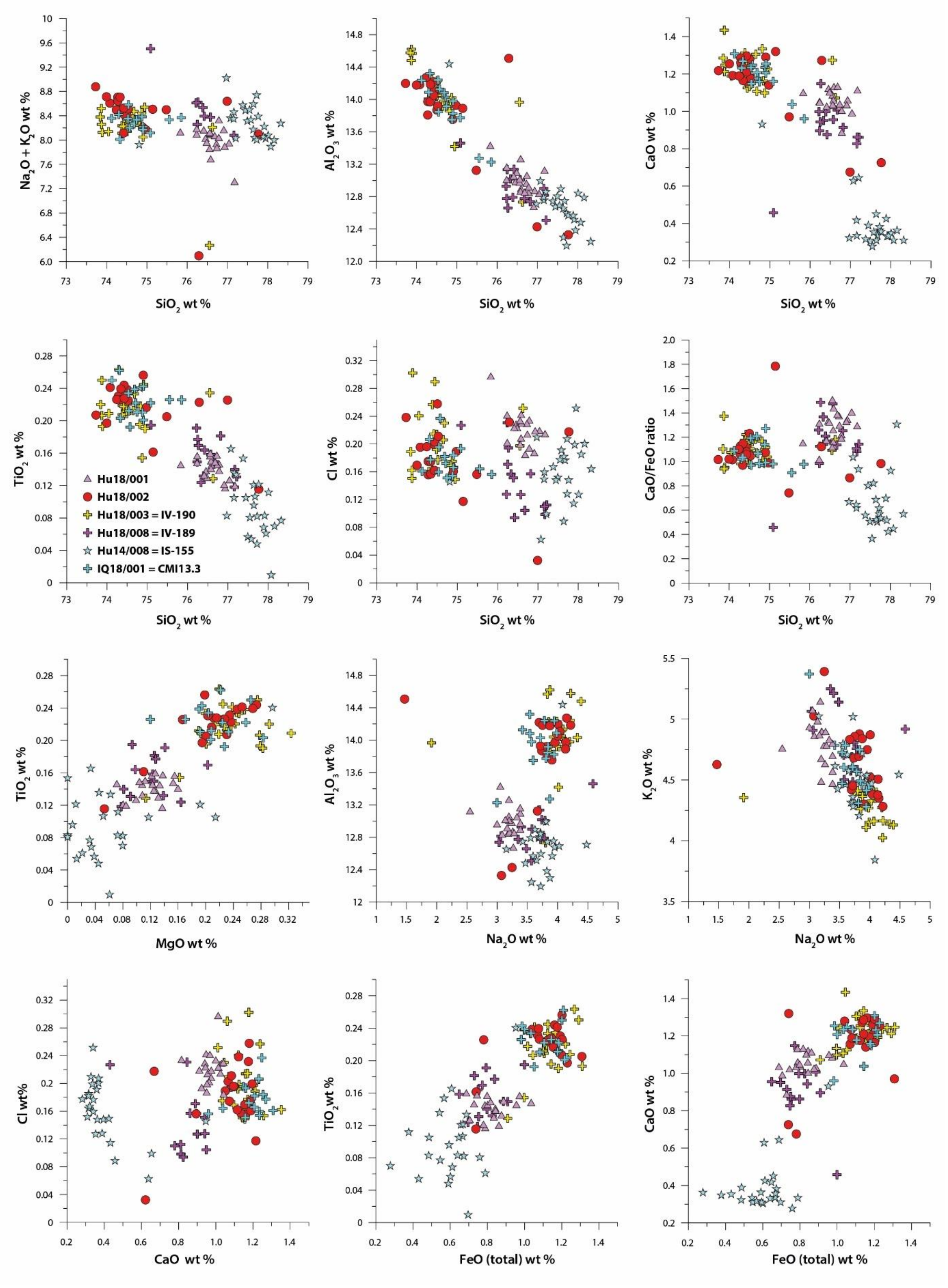

Fig. S3: Harker diagrams of known dated tephra layers and sampled tephra layers from the southern channel (HU18-001, HU18-002). All sampled and reference tephra layers within the study area reveal a distinct rhyolitic composition/origin (>73% SiO_2). Geochemical compositions of measured volcanic glass shard from the two unknown tephra layers within the southern channel, suggest a common origin of HU18-002 to known dated tephra deposits of IV-190 and CMI13.3, verifiable by matching geochemistry. Although HU18-001 also

yield similar geochemical compositions (NaO + K_2O vs. SiO_2, TiO_2 vs FeO, CaO/FeO vs. SiO_2) with known tephra deposits, HU18-001 indicates a much different composition in its Cl vs. CaO ratio. No known and reported tephra and/or volcanic deposit in the vicinity of the study area could be identified.

Cosmogenic Nuclides

Cosmogenic ^{21}Ne concentrations have been measured in 19 samples across three sample sites. Neon compositions of most samples, CREU1[18] intercomparison material, plot within their uncertainties (±1σ) on the spallation line for ^{21}Ne (Fig. S4). Cosmogenic ^{21}Ne concentrations and exposure ages are reported in Table S2.

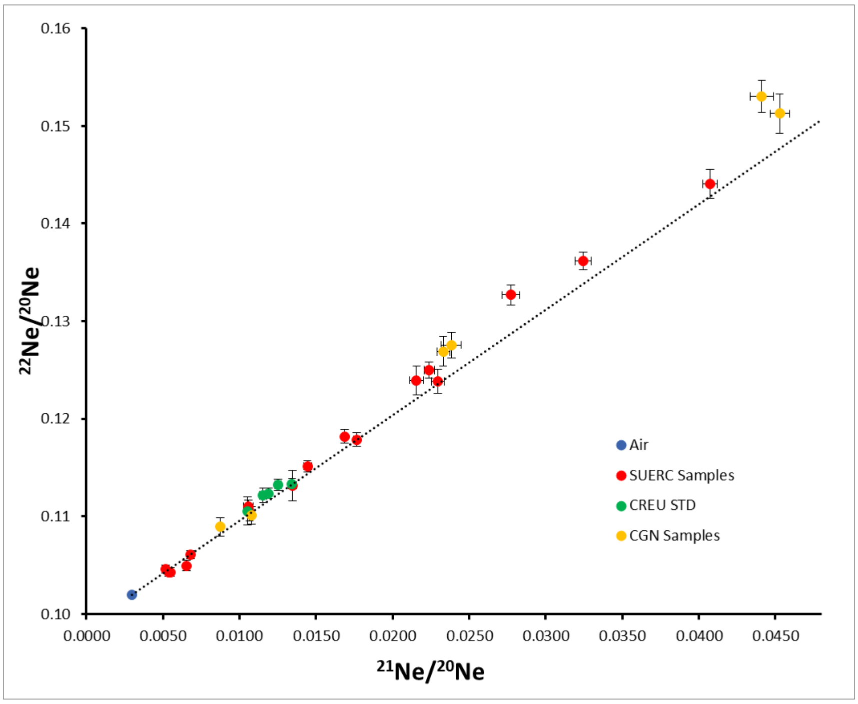

Fig. S4: Neon Triple Isotope Plot ($^{22/20}$Ne vs. $^{21/20}$Ne) for the samples given in Table S2. The dashed line represents a mixture of air and the spallation-derived Ne in quartz[18]. Different colours indicate the air value, samples measured at SUERC, samples measured at Cologne, and CREU standards. Uncertainties are one standard deviation.

Exposure ages (^{21}Ne) range from ~5.6 Ma up to ~35.8 Ma (Fig. S5). The northern (HU15-15) and southern (HU15-18) sampling sites exhibit a clustering of ages between ~6-7 Ma (n=5) and ~6-11 Ma (n=5), respectively. A broad scatter of ages is observed from HU17-001with ages ranging between ~6.5 and ~35.8 Ma. Individual ages are at ~6.5, ~9.6, ~17.1, ~22.8, ~24.9 and at ~35.8 Ma. The latter age is amongst the oldest reported for the Atacama Desert [23-25]. In general, we did not observe any quartz clast with a ^{21}Ne exposure age lower than 5.6 Ma, and the majority of ages are between ~6-13 Ma (Fig. S5).

In nine of the nineteen samples analysed for ^{21}Ne, also ^{10}Be concentrations have been determined. The samples are from all three fan surfaces (Table S2), in all samples ^{10}Be is saturated. At saturation the ^{10}Be concentration becomes time invariant and no further information can be obtained from the sample, than a minimum time/age of exposure required to reach saturation [26]. Samples that are saturated in ^{10}Be indicate a continuous exposure at the surface over at least the last ~5 Myr.

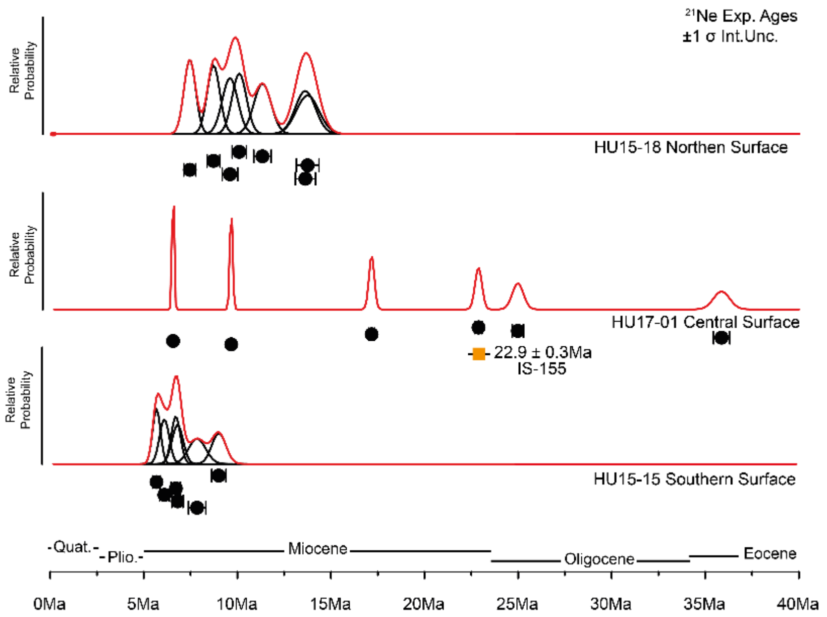

Fig. S5: Cumulative probability density plots of ^{21}Ne ($\pm 1\sigma$ internal uncertainty) of single-clast exposure ages from three fan surfaces. Plots are ordered according to their geographical sampling location from north to south. Note that the central surface reveals a broad distribution of mainly single clasts, however, with two ages at 22.7 and 24.9 Ma. These ages coincide with a tephra layer dated at 22.9±0.3 Ma IS-155,[2] from an exposure close to the surface where it outcrops proximal to the NS reverse reactivated normal fault at the southern clay pan. Surface ages to the north and south reveal a distinct cluster in the Mid to Late Miocene. The data is also presented as points and error bars ($\pm 1\sigma$ internal uncertainty).

Table S2: TCN data (^{21}Ne, ^{10}Be) for surface samples and additional information used to calculate exposure ages. Uncertainties are 1σ.

Sample ID	Neon Isotopes			^{10}Be Isotope	Calculation Information			
	$^{22/20}$Ne	$^{21/20}$Ne	cos. ^{21}Ne atoms/gr	^{10}Be [atoms/gr]	Thickness [cm]	Density	Shielding	Erosionrate
HU15-15A	0.2383 ± 0.0072	0.1164 ± 0.0037	2.23E+08 ± 1.2E+07	1.91E+07 ± 6E+05	0.7	2.65	1	1.74E-06
HU15-15B	0.1043 ± 0.0004	0.0054 ± 0.0001	1.95E+08 ± 8E+06		2	2.65	1	1.74E-06
HU15-15C	0.1043 ± 0.0004	0.0055 ± 0.0001	1.76E+08 ± 7E+06		2	2.65	1	1.74E-06
HU15-15E	0.1179 ± 0.0007	0.0177 ± 0.0002	1.91E+08 ± 6E+06	1.95E+07 ± 6E+05	3	2.65	1	1.74E-06
HU15-15G	0.1061 ± 0.0004	0.0068 ± 0.0001	1.67E+08 ± 6E+06		1	2.65	1	1.74E-06
HU15-15H	0.1046 ± 0.0004	0.0052 ± 0.0000	2.49E+08 ± 9E+06	1.91E+07 ± 6E+05	2	2.65	1	1.74E-06
HU15-18A	0.1182 ± 0.0007	0.0169 ± 0.0002	3.11E+08 ± 1.0E+07		1	2.65	1	1.75E-06
HU15-18B	0.1327 ± 0.0010	0.0277 ± 0.0006	2.32E+08 ± 9E+06		0.7	2.65	1	1.75E-06
HU15-18C	0.1151 ± 0.0006	0.0144 ± 0.0003	1.85E+08 ± 7E+06	1.30E+07 ± 4E+05	1.5	2.65	1	1.75E-06
HU15-18D	0.2608 ± 0.0028	0.1331 ± 0.0024	3.13E+08 ± 1.1E+07		1.1	2.65	1	1.75E-06
HU15-18E	0.1362 ± 0.0009	0.0324 ± 0.0005	2.10E+08 ± 7E+06	1.63E+07 ± 5E+05	2.5	2.65	1	1.75E-06
HU15-18F	0.1250 ± 0.0008	0.0224 ± 0.0003	2.65E+08 ± 9E+06		2	2.65	1	1.75E-06
HU15-18G	0.1441 ± 0.0015	0.0407 ± 0.0005	2.41E+08 ± 8E+06	1.32E+07 ± 4E+05	1.5	2.65	1	1.75E-06
HU17-01A	0.1101 ± 0.0009	0.0108 ± 0.0002	2.38E+08 ± 2E+06	1.59E+07 ± 5E+05	1.5	2.65	1	1.75E-06
HU17-01B	0.1089 ± 0.0010	0.0087 ± 0.0002	1.69E+08 ± 1E+06	1.57E+07 ± 5E+05	1	2.65	1	1.75E-06
HU17-01C	0.1530 ± 0.0017	0.0441 ± 0.0008	6.39E+08 ± 4E+06	1.57E+07 ± 5E+05	0.5	2.65	1	1.75E-06
HU17-01D	0.1269 ± 0.0015	0.0233 ± 0.0004	3.84E+08 ± 2E+06		0.5	2.65	1	1.75E-06
HU17-01E	0.1276 ± 0.0013	0.0238 ± 0.0007	4.93E+08 ± 4E+06		1.5	2.65	1	1.75E-06
HU17-01F	0.1513 ± 0.0020	0.0453 ± 0.0006	4.65E+08 ± 3E+06		1	2.65	1	1.75E-06
Standards Ne								
CREU1 BR3	0.1132 ± 0.0006	0.0125 ± 0.0002	3.66E+08 ± 1.3E+07					
CREU1BR4	0.1133 ± 0.0006	0.0134 ± 0.0001	3.66E+08 ± 1.2E+07					
CREU1 BR5	0.1124 ± 0.0005	0.0119 ± 0.0001	3.61E+08 ± 1.2E+07					
CREU1 BR6	0.1122 ± 0.0007	0.0115 ± 0.0002	3.51E+08 ± 1.4E+07					
CGN0050-CREU	0.1106 ± 0.0014	0.0105 ± 0.0002	3.39E+08 ± 3E+06					

Cosmogenic ^{10}Be and ^{26}Al concentrations have been measured in 29 samples from five sampling sites in channels (Fig. S6, Table S2). ^{26}Al concentrations were measured to detect potential complex exposure histories and verify the ^{10}Be exposure ages. In the following, ages refer to ^{10}Be exposure ages, if not stated otherwise

Clasts from sample HU14-05 (southernmost channel) provide one concordant (i.e., the ^{10}Be and ^{26}Al exposure ages agree within ±2σ, thus lie on the line of continuous exposure; Fig. S6B) exposure age of 636±35 ka (Fig. S6A). The remaining five clasts had a complex exposure history (Fig. S6A).

Four of six quartz clasts from central channel (HU15-16) yield concordant exposure ages. Their probability distribution (Fig. S6A) shows a broad cluster between 730-854 ka (n=3) and one individual age at 1.1 Ma.

Four of five clasts from sample HU17-09 have concordant exposure ages. The lowest ages are close to 820 ka (n=2, 797±34 & 850±36 ka); with additional individual ages at ~1.1 Ma, and ~2.2 Ma (Fig. S6A).

Four of six clasts from (HU15-14) yield concordant ages. Two clasts had a complex exposure history. The two youngest samples have overlapping ages of ~185 ka; additional individual ages are ~332 ka, and ~1.3 Ma (Fig. S6A).

Only one clast of sample HU17-04A has a concordant exposure age of 2.5±0.2 Ma. Two clasts (HU17-04C and D) yield similar ^{10}Be exposure ages, however younger ^{26}Al exposure ages, indicating burial or shallow burial of clasts. In two clasts (HU17-04E and F) the ^{10}Be concentration is saturated, indicating exposure in excess of 5 Ma.

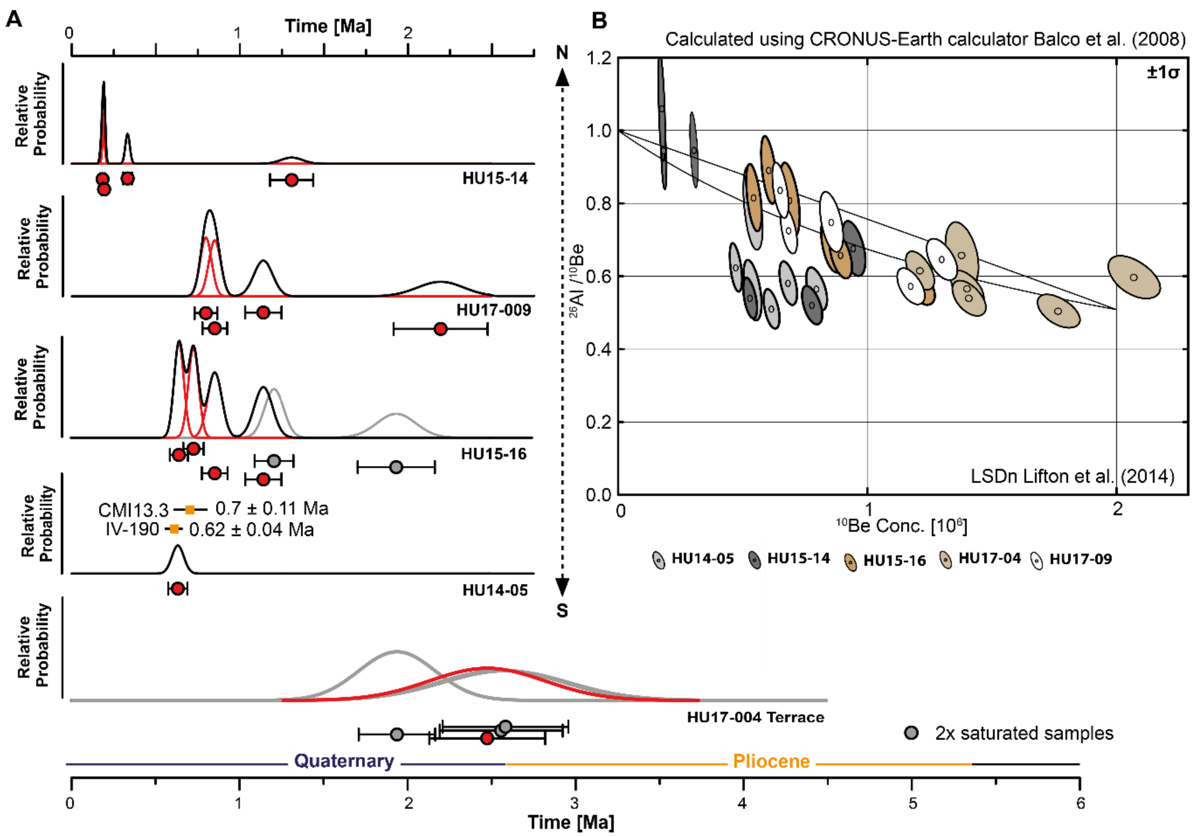

Fig. S6: (A) Cumulative probability density plots of ^{10}Be single-clast exposure ages from four channel beds and one terrace (±1σ). Displayed are ^{10}Be ages, which indicate single-stage exposure histories in red lines/symbols. Grey lines and symbols indicate ^{10}Be ages which do not agree within ±2σ to their corresponding ^{26}Al exposure age. Geochemical fingerprinting of sampled tephra layers in the southern channel indicates that HU18-002 is identical to dated tephra layers (IV-190, CMI13.3, orange squares) that were dated to ~0.6-0.7 Ma [2]. (B) ^{26}Al/^{10}Be vs ^{10}Be concentration plot calculated using LSDn scaling [17] with the Cronus Earth calculator [16] (ellipses are 1sigma). The solid line denotes the 'island of steady erosion' [27]. Continuously exposed samples evolve along the line on top towards the termination at high concentration (saturation in ^{10}Be and ^{26}Al); samples eroding at a steady rate evolve towards discrete points on the lower line. Surface samples that plot below the 'island' had a complex exposure history of intermittent burial and exposure.

Table S3: TCN data for channel samples (^{10}Be, ^{26}Al) and additional information for calculating exposure ages. Uncertainties ±1σ.

Sample ID	^{10}Be Isotope ^{10}Be [atoms/gr]			^{26}Al Isotope ^{26}Al [atoms/gr]			Calculation Information Thickness [cm]	Density	Shielding	Erosionrate
Hu14-05a	3.76E+06	±	1.8E+05	2.06E+07	±	1.7E+06	1	2.65	0.96	1.74E-06
Hu14-05b	3.75E+06	±	1.7E+05	1.47E+07	±	1.3E+06	1	2.65	0.96	1.74E-06
HU14-05c	3.26E+06	±	1.1E+05	1.43E+07	±	9E+05	2	2.65	0.96	1.74E-06
HU14-05d	5.70E+06	±	2.0E+05	2.17E+07	±	1.3E+06	2	2.65	0.96	1.74E-06
HU14-05e	4.17E+06	±	1.4E+05	1.50E+07	±	9E+05	3	2.65	0.96	1.74E-06
HU14-05f	4.67E+06	±	1.6E+05	1.86E+07	±	1.1E+06	4	2.65	0.96	1.74E-06
HU15-014a	1.38E+06	±	5E+04	8.96E+06	±	5.7E+05	1	2.65	0.96	1.74E-06
HU15-014b	1.28E+06	±	7E+04	9.74E+06	±	8.04E+05	2	2.65	0.96	1.74E-06
HU15-014c	2.32E+06	±	8E+04	1.52E+07	±	9.7E+05	2	2.65	0.96	1.74E-06
HU15-014d	7.48E+06	±	2.5E+05	3.39E+07	±	2.3E+06	3	2.65	0.96	1.74E-06
HU15-014e	3.94E+06	±	1.4E+05	1.50E+07	±	8.9E+05	3	2.65	0.96	1.74E-06
HU15-014f	5.94E+06	±	2.1E+05	2.05E+07	±	1.2E+06	4	2.65	0.96	1.74E-06
HU15-016b	6.57E+06	±	2.3E+05	3.02E+07	±	2.0E+06	1.5	2.65	0.96	1.74E-06
HU15-016c	5.01E+06	±	2.0E+05	2.81E+07	±	2.1E+06	2.5	2.65	0.96	1.74E-06
HU15-016d	9.40E+06	±	3.2E+05	3.64E+07	±	2.0E+06	3	2.65	0.96	1.74E-06
HU15-016e	6.80E+06	±	2.3E+05	2.98E+07	±	1.7E+06	3	2.65	0.96	1.74E-06
HU15-016f	3.94E+06	±	1.5E+05	2.24E+07	±	1.5E+06	2.5	2.65	0.96	1.74E-06
HU15-016g	4.28E+06	±	1.5E+05	2.65E+07	±	1.6E+06	4	2.65	0.96	1.74E-06
HU17-004a	9.03E+06	±	3.0E+05	4.10E+07	±	3.6E+06	1.25	2.65	0.96	1.76E-06
HU17-004b	8.01E+06	±	2.6E+05	3.37E+07	±	1.7E+06	0.25	2.65	0.96	1.76E-06
HU17-004c	9.18E+06	±	3.0E+05	3.58E+07	±	1.9E+06	1	2.65	0.96	1.76E-06
HU17-004d	9.23E+06	±	3.1E+05	3.41E+07	±	1.8E+06	1	2.65	0.96	1.76E-06
HU17-004e	1.15E+07	±	4E+05	4.04E+07	±	2.2E+06	0.75	2.65	0.96	1.76E-06
HU17-004f	1.26E+07	±	4E+05	5.41E+07	±	3.0E+06	0.75	2.65	0.96	1.76E-06
HU17-009b	9.89E+06	±	3.3E+05	4.38E+07	±	2.3E+06	1	2.65	0.96	1.74E-06
HU17-009c	4.84E+06	±	1.6E+05	2.41E+07	±	1.2E+06	1.9	2.65	0.96	1.74E-06
HU17-009d	8.92E+06	±	2.9E+05	3.42E+07	±	1.8E+06	1.4	2.65	0.96	1.74E-06
HU17-009e	4.58E+06	±	1.6E+05	2.65E+07	±	1.4E+06	1.45	2.65	0.96	1.74E-06
HU17-009f	6.38E+06	±	2.3E+05	3.26E+07	±	2.1E+06	0.4	2.65	0.96	1.74E-06

Table S4: Compilation of TCN exposure ages. Given are internal and external uncertainties as calculated by "The online exposure age calculator formerly known as the CRONUS-Earth online exposure age calculator." Version 3, http://hess.ess.washington.edu/math/v3/v3_age_in.html; [16].

Sample ID	^{21}Ne [Ma]	±1σ	Int. Unc.	Ext. Unc.	^{10}Be [Ma]
HU15-15A	7.78	±	0.46	0.71	saturated
HU15-15B	6.73	±	0.30	0.55	
HU15-15C	6.02	±	0.26	0.48	
HU15-15E	6.64	±	0.24	0.52	saturated
HU15-15G	5.61	±	0.21	0.43	
HU15-15H	8.93	±	0.38	0.74	saturated
HU15-18A	13.52	±	0.54	1.16	
HU15-18B	9.49	±	0.42	0.80	
HU15-18C	7.35	±	0.31	0.60	saturated
HU15-18D	13.64	±	0.60	1.20	
HU15-18E	8.61	±	0.34	0.70	saturated
HU15-18F	11.23	±	0.46	0.95	
HU15-18G	9.99	±	0.38	0.82	saturated
HU17-01A	9.56	±	0.07	0.69	saturated
HU17-01B	6.46	±	0.05	0.44	saturated
HU17-01C	35.76	±	0.44	3.58	saturated
HU17-01D	17.06	±	0.14	1.38	
HU17-01E	24.87	±	0.30	2.49	
HU17-01F	22.77	±	0.18	2.01	

Sample ID	^{10}Be [ka]	±1σ	Int. Unc.	Ext. Unc.	^{26}Al [ka]	±1σ	Int. Unc.	Ext. Unc.
Hu14-05a	636	±	35	57	553	±	63	90
Hu14-05b	633	±	35	56	360	±	39	54
HU14-05c	543	±	22	43	349	±	26	45
HU14-05d	1033	±	48	94	599	±	50	87
HU14-05e	745	±	32	62	378	±	29	49
HU14-05f	846	±	37	73	506	±	39	69
HU15-014a	192	±	8	14	189	±	13	22
HU15-014b	183	±	10	15	205	±	19	27
HU15-014c	332	±	13	25	340	±	26	43
HU15-014d	1308	±	64	128	1024	±	121	196
HU15-014e	621	±	26	50	337	±	24	42
HU15-014f	1002	±	46	90	520	±	40	71
HU15-016b	1144	±	55	108	892	±	94	156
HU15-016c	855	±	42	77	817	±	96	145
HU15-016d	1933	±	116	230	1200	±	130	240
HU15-016e	1207	±	57	115	890	±	80	148
HU15-016f	642	±	29	54	588	±	52	87
HU15-016g	728	±	31	61	778	±	71	124
HU17-004a	2472	±	168	344	2466	±	980	1361
HU17-004b	1935	±	110	227	1386	±	152	302
HU17-004c	2555	±	178	366	1602	±	207	403
HU17-004d	2581	±	184	374	1445	±	174	331
HU17-004e	saturated				2289	±	485	916
HU17-004f	saturated				saturated			
HU17-009b	2194	±	137	280	1864	±	288	556
HU17-009c	850	±	36	73	690	±	52	100
HU17-009d	1834	±	102	209	1125	±	107	210
HU17-009e	797	±	34	68	788	±	65	123
HU17-009f	1139	±	56	108	1031	±	116	195

Topographic Data
Swath Profiles

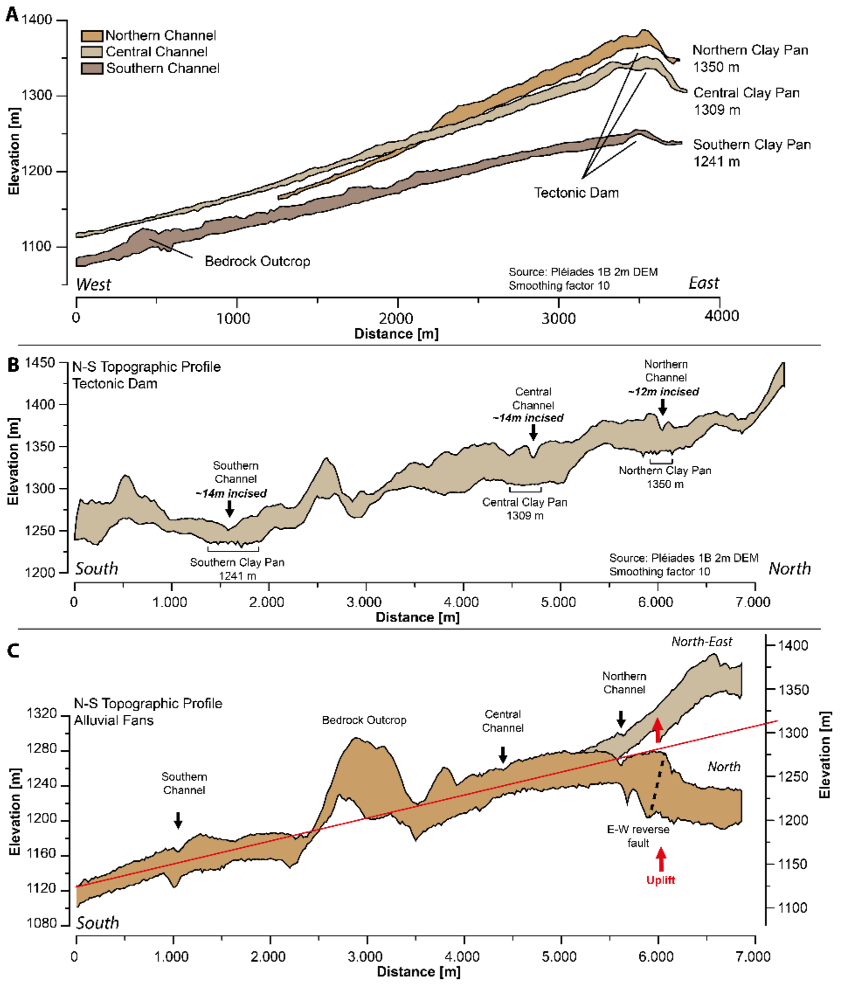

Fig. S7: Topographic swath profiles from the study area based on Pléiades 1B (Fig. S1). Shown are maximum and minimum elevations. (A) Channel swath profiles of the southern, central and northern channel. Note the effect of the northern E-W reverse fault causing higher altitudes towards the northern channel. (B) Swath profiles over the NS running reverse fault covering the tectonic dam area and parts of the clay pan systems to the east. (C) Swath profile running from south to north over the Pampa de la Perdiz, with a branch in the northern segment to the NE towards the northernmost clay pan.

Sinuosity

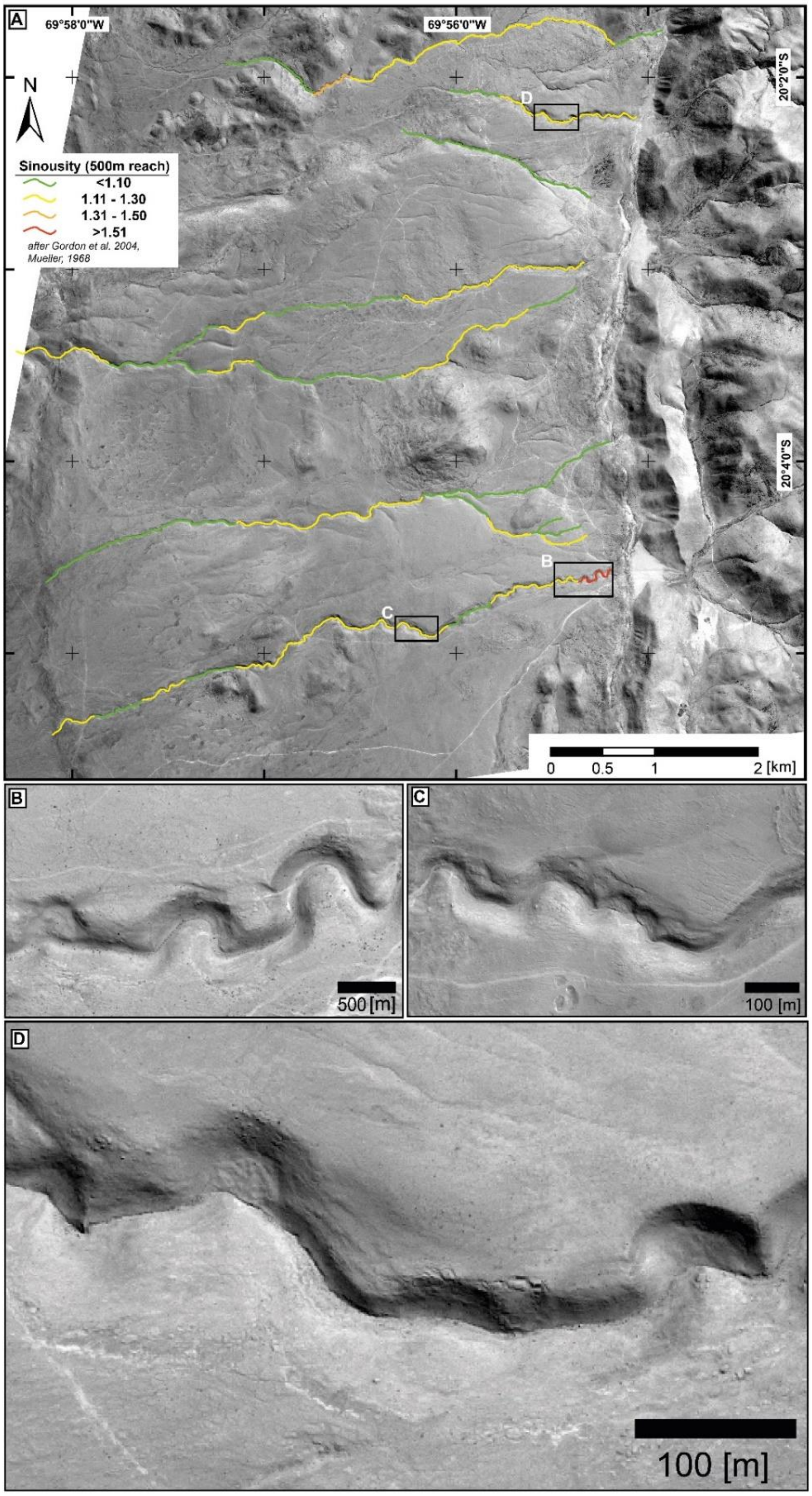

Fig. S8: Pléiades 1B panchromatic satellite imagery from the Huara site. (A) Map with calculated sinuosities for 500m moving windows along the fluvial channels, using the classifications of [28,29]*. Black boxes indicate locations of frames (B-E). (B) High sinuosity channel with steep slopes. Toe-slope erosion and/or earthquakes caused mass failures partly obliterate the initial meander pattern. (C) Slope mass failures due to oversteepening and toe-slope erosion. Large cracks in the crusts presumably formed by gravitational forcing. (D) Mass failures at the rims of the channel. The blocky nature of the failures highlights the cohesion of the* $CaSO_4$*-rich soils and illustrate their thickness.*

5. Chronological constraints on landscape evolution

Chronology of depositional environments

In situ cosmogenic nuclide exposure dating became a widely accepted and applied tool to study landscape evolution in the Atacama Desert, e.g., timing of sediment deposition, abandonment of surfaces or fluvial incision [23-25,30-33]. One of the main challenges of dating old surfaces in arid areas, such as the Atacama Desert, with generally low surface activity is the assessment of potential pre-exposure, so-called inheritance, of cosmogenic nuclides during initial erosion, transport and final deposition. Likewise, the effect of post-depositional erosion/exhumation has to be considered. Both processes, pre-exposure and post-depositional exhumation, will lead to either erroneously old or young exposure ages. Multiple single clast dating from depositional surfaces can be applied to gain knowledge about the potential influence of these effects to the dataset. Tight clusters of ages that overlap within their uncertainties would be expected if pre-exposure is negligible compared to the final exposure. Significant pre-exposure of individual clasts introduces a random scatter that renders calculated ages higher. The lowest age of a stable surface with material with significant pre-exposure provides the maximum exposure age of the surface. Post-depositional exhumation of clasts introduces a random scatter that renders calculated ages younger. If significant pre-exposure can be excluded, the oldest age from a surface clast would provide a minimum age of an eroding surface. If significant pre-exposure and exhumation of clast cannot be excluded, the age of the surface may not be constrained by the calculated exposure ages. Independent geological and geochronological evidence can be used to constrain likely levels of pre-exposure.

A dispersal towards younger ages could be predicted from erosion of low-angle surfaces, as the resulting lag-deposit is built up from material from various depths of shielding. When significant pre-exposure can be neglected, the oldest ages, highest concentration of a cosmogenic nuclide, derived for clasts on such a surface are the best estimate for their minimum deposition age. Given the close proximity, small size and steepness of the catchments that provided the sediments of the alluvial fan surfaces studied, relatively fast erosion (fast compared to the later exposure) and short transit times may be assumed, rendering a significant pre-exposure unlikely.

Additional site-specific information may be extracted from the tephra layers that are interbedded in the alluvial deposits, which provide maximum exposure ages of the overlying surfaces. For instance, the tephra layer near the top of the main alluvial fan surface provides an age of 22.9±0.3 Ma (IS 155, Ar/Ar, biotite, [2]). The age lends credence to the surface exposure ages of similar and lower ages we find further downstream on the fan surface (sample HU 15-1). Early- to Mid-Miocene surfaces are common in the hyperarid core of the Atacama Desert, [23,24].

Chronology of fluvial features

Interpretation of cosmogenic nuclide ages from channel beds is generally subject to the same difficulties as for fan surfaces, i.e., pre-exposure or erosion. Transported and deposited clasts within incising channel beds could originate from two different populations/origins, either (1) from eroded and transported material sourced in the catchment, or (2) from lateral erosion of unconsolidated alluvium within the channel. Lateral channel erosion could re-expose previous and/or partly shielded clasts, which could have a significant cosmogenic inventory from pre-exposure from depth dependent accumulation of cosmogenic nuclides within the incised alluvium. Due to the large age of the fan deposits studied here (>5 Ma, see above) we can exclude a pre-exposure signal from the initial erosion and transport, inventories of ^{10}Be and ^{26}Al from the initial would have decayed to insignificant levels.

References

1 Riquelme, R., Martinod, J., Herail, G., Darrozes, J. & Charrier, R. A geomorphological approach to determining the Neogene to Recent tectonic deformation in the Coastal Cordillera of northern Chile (Atacama). *Tectonophysics* **361**, 255-275, doi:10.1016/s0040-1951(02)00649-2 (2003).

2 Vásquez, P. & Sepúlveda, F. Cartas Iquique y Pozo Almonte - Región de Tarapacá No. 161-163 Escala 1:100.000. *Carta Geológica de Chile Serie Geología Básica* (2013).

3 Mortimer, C. & Saric, N. Landform evolution in the coastal region of Tarapacá Province, Chile. *Revue de géomorphologie dynamique* **21**, 162-170 (1972).

4 Wells, S. G., McFadden, L. D., Poths, J. & Olinger, C. T. Cosmogenic ^{3}He surface exposure dating of stone pavements. *Geology* **23**, 613-616 (1995).

5 Wang, F. *et al.* Beryllium-10 concentrations in the hyper-arid soils in the Atacama Desert, Chile: Implications for arid soil formation rates and El Niño driven changes in Pliocene precipitation. *Geochimica et Cosmochimica Acta* **160**, 227-242, doi:http://dx.doi.org/10.1016/j.gca.2015.03.008 (2015).

6 Leopold, L. B., Wolman, M. G. & Miller, J. P. *Fluvial processes in geomorphology*. (Courier Corporation, 1995).

7 Kuehn, S. C., Froese, D. G., Shane, P. A. & Participants, I. I. The INTAV intercomparison of electron-beam microanalysis of glass by tephrochronology laboratories: results and recommendations. *Quaternary International* **246**, 19-47 (2011).

8 Jochum, K. P. *et al.* MPI-DING reference glasses for in situ microanalysis: New reference values for element concentrations and isotope ratios. *Geochemistry, Geophysics, Geosystems* **7** (2006).

9 Kohl, C. & Nishiizumi, K. Chemical isolation of quartz for measurement of in-situ-produced cosmogenic nuclides. *Geochimica et Cosmochimica Acta* **56**, 3583-3587, doi:doi.org/10.1016/0016-7037(92)90401-4 (1992).

10 Binnie, S. A. *et al.* Separation of Be and Al for AMS using single-step column chromatography. *Nuclear Instruments and Methods in Physics Research Section B: Beam Interactions with Materials and Atoms* (2015).

11 Stone, J. *et al.* Co-precipitated silver–metal oxide aggregates for accelerator mass spectrometry of 10Be and 26Al. *Nuclear Instruments and Methods in Physics Research Section B: Beam Interactions with Materials and Atoms* **223**, 272-277 (2004).

12 Dewald, A. *et al.* CologneAMS, a dedicated center for accelerator mass spectrometry in Germany. *Nuclear Instruments & Methods in Physics Research Section B-Beam Interactions with Materials and Atoms* **294**, 18-23, doi:10.1016/j.nimb.2012.04.030 (2013).

13 Nishiizumi, K. *et al.* Absolute calibration of Be-10 AMS standards. *Nucl. Instr. Meth. Phys. Res. B* **258**, 403-413 (2007).
14 Nishiizumi, K. Preparation of 26 Al AMS standards. *Nuclear Instruments and Methods in Physics Research Section B: Beam Interactions with Materials and Atoms* **223**, 388-392 (2004).
15 Binnie, S. A. *et al.* Preliminary results of CoQtz-N: A quartz reference material for terrestrial in-situ cosmogenic 10Be and 26Al measurements. *Nuclear Instruments and Methods in Physics Research Section B: Beam Interactions with Materials and Atoms* **456**, 203-212 (2019).
16 Balco, G., Stone, J. O., Lifton, N. A. & Dunai, T. J. A complete and easily accessible means of calculating surface exposure ages or erosion rates from (10)Be and (26)Al measurements. *Quaternary Geochronology* **3**, 174-195, doi:10.1016/j.quageo.2007.12.001 (2008).
17 Lifton, N., Sato, T. & Dunai, T. J. Scaling in situ cosmogenic nuclide production rates using analytical approximations to atmospheric cosmic-ray fluxes. *Earth and Planetary Science Letters* **386**, 149-160, doi:10.1016/j.epsl.2013.10.052 (2014).
18 Vermeesch, P. *et al.* Interlaboratory comparison of cosmogenic Ne-21 in quartz. *Quaternary Geochronology* **26**, 20-28, doi:doi.org/10.1016/j.quageo.2012.11.009 (2015).
19 Ma, Y. & Stuart, F. M. The use of in-situ cosmogenic 21 Ne in studies on long-term landscape development. *Acta Geochimica*, 1-13 (2017).
20 Codilean, A. T. *et al.* Single-grain cosmogenic ^{21}Ne concentrations in fluvial sediments reveal spatially variable erosion rates. *Geology* **36**, 159-162 (2008).
21 Ritter, B., Vogt, A. & Dunai, T. J. Technical Note: Noble gas extraction procedure and performance of the Cologne Helix MC Plus multi-collector noble gas mass spectrometer for cosmogenic neon isotope analysis. *Geochronology* **2021** (2021).
22 Borchers, B. *et al.* Geological calibration of spallation production rates in the CRONUS-Earth project. *Quaternary Geochronology* **31**, 188-198 (2016).
23 Ritter, B. *et al.* Neogene fluvial landscape evolution in the hyperarid core of the Atacama Desert. *Scientific Reports* **8**, 13952, doi:10.1038/s41598-018-32339-9 (2018).
24 Dunai, T. J., Lopez, G. A. G. & Juez-Larre, J. Oligocene-Miocene age of aridity in the Atacama Desert revealed by exposure dating of erosion-sensitive landforms. *Geology* **33**, 321-324, doi:doi.org/10.1130/g21184.1 (2005).
25 Carrizo, D., Gonzalez, G. & Dunai, T. Neogene constriction in the northern Chilean Coastal Cordillera: Neotectonics and surface dating using cosmogenic 21Ne. *Revista Geologica De Chile* **35**, 1-38 (2008).
26 Dunai, T. J. *Cosmogenic Nuclides: Principles, concepts and applications in the Earth surface sciences*. (Cambridge University Press, 2010).
27 Lal, D. Cosmic ray labeling of erosion surfaces: in situ nuclide production rates and erosion models. *Earth Planet. Sci. Lett.* **104**, 424-439 (1991).
28 Gordon, N. D., McMahon, T. A., Finlayson, B. L., Gippel, C. J. & Nathan, R. J. *Stream hydrology: an introduction for ecologists*. (John Wiley and Sons, 2004).
29 Mueller, J. E. An introduction to the hydraulic and topographic sinuosity indexes. *Annals of the association of american geographers* **58**, 371-385 (1968).
30 Evenstar, L. A. *et al.* Multiphase development of the Atacama Planation Surface recorded by cosmogenic 3He exposure ages: Implications for uplift and Cenozoic climate change in western South America. *Geology* **37**, 27-30, doi:10.1130/g25437a.1 (2009).
31 Evenstar, L. *et al.* Geomorphology on geologic timescales: Evolution of the late Cenozoic Pacific paleosurface in Northern Chile and Southern Peru. *Earth-Science Reviews* (2017).
32 Binnie, S. *et al.* The origins and implications of paleochannels in hyperarid, tectonically active regions: The northern Atacama Desert, Chile. *Global and Planetary Change* **185**, 103083, doi:doi.org/10.1016/j.gloplacha.2019.103083 (2020).
33 Ritter, B., Binnie, S. A., Stuart, F. M., Wennrich, V. & Dunai, T. J. Evidence for multiple Plio-Pleistocene lake episodes in the hyperarid Atacama Desert. *Quaternary Geochronology* **44**, 1-12, doi:doi.org/10.1016/j.quageo.2017.11.002 (2018).
